# Correction: Study protocol of the ASTOP trial: A multicenter, randomized, double-blind, placebo-controlled trial of presurgical aspirin administration for the prevention of thromboembolic complications of coil embolization for ruptured aneurysms

**DOI:** 10.1371/journal.pone.0329657

**Published:** 2025-08-04

**Authors:** Sakyo Hirai, Kyohei Fujita, Shoko Fujii, Satoru Takahashi, Keigo Shigeta, Jun Karakama, Yukiko Enomoto, Yohei Sato, Masataka Yoshimura, Shin Hirota, Tatsuya Mizoue, Yoshikazu Yoshino, Yoshihisa Kawano, Toshihiro Yamamura, Shinya Kohyama, Masaru Hirohata, Shinichi Yoshimura, Yosuke Ishii, Toshihiro Yamauchi, Naoki Taira, Yoshiki Obata, Makoto Sakamoto, Masato Inoue, Motoshige Yamashina, So Tokunaga, Toshio Higashi, Kana Sawada, Hidetoshi Mochida, Keisuke Ido, Masataka Takeuchi, Tomoji Takigawa, Yasushi Takagi, Masafumi Morimoto, Masataka Nanto, Kazunori Miki, Kouichi Misaki, Koichi Arimura, Yoshiki Hanaoka, Mutsuya Hara, Shoko Hara, Kota Yokoyama, Jun Ooyama, Ryoichi Hanazawa, Hiroyuki Sato, Akihiro Hirakawa, Megumi Ishiguro, Shigeru Nemoto, Kazutaka Sumita

The hyperlink in the Trial Registration section is incorrect. The correct hyperlink is: https://jrct.mhlw.go.jp.
