# Supplementary material for: Study protocol of the ASTOP trial: A multicenter, randomized, double-blind, placebo-controlled trial of presurgical aspirin administration for the prevention of thromboembolic complications of coil embolization for ruptured aneurysms
Source: PLoS One. 2024 Sep 26;19(9):e0310906. doi: 10.1371/journal.pone.0310906 (PMC11426478; doi:10.1371/journal.pone.0310906)
Supplement: S2 File — (DOCX) [file pone.0310906.s002.docx]

**Study protocol of the ASTOP trial:**

**A multicenter, randomized, double-blind, placebo-controlled trial of**

**pre-surgical aspirin administration for the prevention of thromboembolic complications of coil embolization for ruptured aneurysms**

(ASTOP study)

jRCT Registry Number: jRCTs031210421

◆Principal investigator: Kazutaka Sumita

Department of Endovascular surgery, Tokyo Medical and Dental University Hospital

Address: 1-5-45 Yushima, Bunkyo-ku, Tokyo 113-8519

Telephone number: 03-3813-6111 FAX: 03-5803-0110

E-mail: sumita.nsrg@tmd.ac.jp

◆Date of preparation, date of approval, and date of revision

The first edition of the protocol on August 18, 2021: Approved by the Certified Review Board of the Tokyo Medical and Dental University on September 28, 2021

The second edition of the protocol on November 11, 2021: Approved by the Certified Review Board of the Tokyo Medical and Dental University on November 26, 2021

The third edition of the protocol on November 25, 2021: Approved by the Certified Review Board of the Tokyo Medical and Dental University on December 22, 2021

The 4th edition of the protocol on January 31, 2022: Approved by the Certified Review Board of the Tokyo Medical and Dental University on February 18, 2022

The 5th edition of the protocol on February 18, 2022: Approved by the Certified Review Board of the Tokyo Medical and Dental University on March 7, 2022

The 6th edition of the protocol on March 11, 2022 Approved by the Certified Review Board of the Tokyo Medical and Dental University on March 24, 2022

The 7th edition of the protocol on March 24, 2022: Approved by the Certified Review Board of the Tokyo Medical and Dental University on April 22, 2022

The 8th edition of the protocol on April 20, 2022. Approved by the Certified Review Board of the Tokyo Medical and Dental University on May 20, 2022

The 9th edition of the protocol on May 6, 2022: Approved by the Certified Review Board of the Tokyo Medical and Dental University on May 20, 2022

The 10th edition of the protocol on June 6, 2022 Approved by the Certified Review Board of the Tokyo Medical and Dental University on June 17, 2022

The 11th edition of the protocol on June 28, 2022: Approved by the Certified Review Board of the Tokyo Medical and Dental University on July 22, 2022

The 12th edition of the protocol on July 28, 2022 Approved by the Certified Review Board of the Tokyo Medical and Dental University on August 19, 2022

The 13th edition of the protocol on August 31, 2022 Approved by the Certified Review Board of the Tokyo Medical and Dental University on September 16, 2022

The 14th edition of the protocol on September 30, 2022 Approved by the Certified Review Board of the Tokyo Medical and Dental University on October 21, 2022

The 15th edition of the protocol on November 4, 2022: Approved by the Certified Review Board of the Tokyo Medical and Dental University on November 22, 2023

The 16th edition of the protocol on March 27, 2023: Approved by the Certified Review Board of the Tokyo Medical and Dental University on May 26, 2023

The 17th edition of the protocol on June 23, 2023: Approved by the Certified Review Board of the Tokyo Medical and Dental University on July 21, 2023

The 18th edition of the protocol on October 18, 2023 Approved by the Certified Review Board of the Tokyo Medical and Dental University on November 17, 2023

The 19th edition of the protocol on January 19, 2024 Approved by the Certified Review Board of the Tokyo Medical and Dental University on Feburary 16, 2024

The 20th edition of the protocol on Feburary 16, 2024 Approved by the Certified Review Board of the Tokyo Medical and Dental University on March 26, 2024

**List of Abbreviations**

| Abbreviation |  |
| --- | --- |
| ACT | Activating Coagulation Time |
| ASA | Acetylsalicylic acid |
| BAT | Balloon assist technique |
| CT | Computed Tomography |
| CTA | Computed Tomography Angiography |
| DAC | Distal access catheter |
| DCT | Double catheter technique |
| DWI | Diffusion wighted imaging |
| INV | Investigator |
| IRC | Independent Review Committee |
| JSNET | The Japanese society for Neuroendovascular therapy |
| MRA | Magnetic Resonance Angiography |
| MRI | Magnetic Resonance Imaging |
| mRS | Modified Rankin Scale |
| RCT | Randomized Control Trial |
| SAH | Subarachnoid hemorrhage |
| SAT | Stent assist technique |
| WFNS | World Federation of Neurosurgical Societies |

Table of contents

[**<Overview>** 5](#_Toc169183367)

[**1. Study Background** 11](#_Toc169183368)

[**2. Purpose of the study** 12](#_Toc169183369)

[**3. Summary of Drugs Used in Clinical Research** 12](#_Toc169183370)

[**4. Patient population** 13](#_Toc169183371)

[4-1. Inclusion criteria 13](#_Toc169183372)

[4-2. Exclusion criteria 13](#_Toc169183373)

[**5. Method of obtaining consent from the patients** 14](#_Toc169183374)

[**6. Methods of Research (Content)** 14](#_Toc169183375)

[6-1. Type and design of studies 16](#_Toc169183376)

[6-2. Study period 16](#_Toc169183377)

[6-3. Dosage and administration and duration of administration of the research drug 17](#_Toc169183378)

[6-4. Registration and allocation method 17](#_Toc169183379)

[6-5. Response after completion of study 18](#_Toc169183380)

[**7. Endpoint** 18](#_Toc169183381)

[7-1. Items related to the evaluation of efficacy 18](#_Toc169183382)

[7-2. Items related to safety evaluation 19](#_Toc169183383)

[**8. Observation and examination items** 22](#_Toc169183384)

[**9. Discontinuation criteria** 31](#_Toc169183385)

[9-1. Discontinuation within time from post-registration allocation to embolization 31](#_Toc169183386)

[9-2. Discontinuation during period from embolization to 3 months after surgery 31](#_Toc169183387)

[**10. Handling of adverse events** 31](#_Toc169183388)

[10-1. Response to patients when adverse events occur 31](#_Toc169183389)

[10-2. Reports on outbreaks of diseases 31](#_Toc169183390)

[**11. Handling of the occurrence of non-compliance with the research protocol** 32](#_Toc169183391)

[**12. Reporting and methods to administrators of medical institutions** 32](#_Toc169183392)

[**13. Study completion, discontinuation, or interruption** 33](#_Toc169183393)

[13-1. Completion of the study 33](#_Toc169183394)

[13-2. Discontinuation or interruption of research 33](#_Toc169183395)

[**14. Statistical matter** 33](#_Toc169183396)

[14-1. Full analysis set 34](#_Toc169183397)

[14-2. Number of patients planned and rationale 34](#_Toc169183398)

[14-3. Statistical analysis 34](#_Toc169183399)

[**15. Monitoring** 35](#_Toc169183400)

[**16 Ethics** 36](#_Toc169183401)

[16-1. Declaration of Helsinki and the Clinical Research Act. 36](#_Toc169183402)

[16-2. Consideration for human rights (protection of privacy) 36](#_Toc169183403)

[16-3. Consideration for safety and disadvantage 36](#_Toc169183404)

[**17. Cost burden for the study participants** 36](#_Toc169183405)

[**18. Health Damage Compensation and Insurance Subscription** 36](#_Toc169183406)

[18-1. Compensation for health damage 36](#_Toc169183407)

[18-2. Purchase of compensation insurance and liability insurance 36](#_Toc169183408)

[**19. Handling of Personal Information** 37](#_Toc169183409)

[**20. Storage of records** 37](#_Toc169183410)

[**21. Enrollment of study plans and publication of study results** 37](#_Toc169183411)

[**22. System for conducting research** 37](#_Toc169183412)

[**23. Research funding and conflicts of interest** 41](#_Toc169183413)

[**24. Changes in the research protocol** 41](#_Toc169183414)

[**25. Reference and reference lists** 41](#_Toc169183415)

# **<Overview>**

| **Study title** | Pre-surgical Aspirin Administration for the Prevention of Thromboembolic Complications of Coil Embolization for Ruptured Aneurysms  (ASTOP study) |
| --- | --- |
| **Purpose of the study** | During endovascular treatment of ruptured cerebral aneurysms, thromboembolisms are known as uncontrollable therapeutic complications, and further improvement of treatment outcomes can be expected by controlling these complications. We hypothesized that the administration of preoperative antiplatelet agents for endovascular treatment may reduce thromboembolic complications.  The patients who are planned to undergo endovascular treatment for acute ruptured saccular cerebral aneurysms will be randomly assigned to receive aspirin or placebo preoperatively. The incident rate of intraoperative thromboembolic events, postoperative new neurological dysfunction, new cerebral infarction in postoperative magnetic resonance imaging (MRI), perioperative cerebral ischemic events or all hemorrhagic events, and mRS at 3 months will be compared between the two groups. |
| **Study Design** | A multicenter, randomized, double-blind, placebo-controlled study |
| **Phase** | Confirmatory study |
| **Test drug** | Generic name: Aspirin  Dosage form: Powder  Storage conditions: Storage at room temperature |
| **Inclusion criteria** | Patients who meet all of the following criteria   1. Patients with ruptured cerebral aneurysms in the acute phase 2. Patients scheduled for aneurysm embolization within 72 h of onset 3. Patients aged 20 years or older at the time of presentation 4. Patients for whom written informed consent has been obtained from the person or their legally acceptable representative (e.g., spouse, parent, adult child) for participation in the study. |
| **Exclusion criteria** | Patients who meet any of the following criteria   1. Pre-stroke mRS ≧ 4 2. Patients with dissecting cerebral aneurysms in whom parent vessel occlusion was attempted 3. Patients with ruptured recurrent aneurysms after cerebral aneurysm coil embolization 4. Patients with ruptured cerebral aneurysms associated with cerebral arteriovenous malformations, moyamoya disease, or infectious cerebral aneurysms 5. Patients treated simultaneously for two or more aneurysms 6. Patients already taking antiplatelet drugs 7. Patients with a history of allergy to lactose, aspirin, or salicylic acid preparations 8. Patients who cannot undergo MRI 9. Patients with contraindications to aspirin 10. Patients who are considered inappropriate for participation in the study by the treating physician |
| **Endpoint** | **Primary outcomes**   1. Incidence rate of intraoperative thromboembolic complications 2. Incidence rate of symptomatic ischemic lesions on MRI diffusion-weighted imaging (DWI) evaluated by Independent Review Committee (IRC)   **Key secondary outcomes**   1. Incidence rate of all bleeding events within 14 days of enrollment 2. Incidence rate of cerebral ischemic events in the first 14 days after MRI   **Other secondary outcome**   1. Incidence rate of symptomatic ischemic lesions in MRI-DWI evaluated by INV 2. mRS score at 3 months   **Exploratory outcomes**   1. Number and size of ischemic lesions in MRI-DWI evaluated by the IRC and INV 2. Incidence rate of cerebral hemorrhage along the tube tract for ventricular drainage on computed tomography (CT) scans in the first 14 days after endovascular treatment (EVT). |
| **Research methods** | Patients who are scheduled for coil embolization for a single intracranial aneurysm, presumed to be the rupture site, within 72 h of the onset of subarachnoid hemorrhage and have provided written informed consent to participate in this study will be divided into the following two groups using an Interactive Web Response System (IWRS).  [Aspirin group].  Aspirin 200mg will be administered via a nasogastric tube after induction of general anesthesia prior to embolization.  [Placebo group].  Placebo (lactose) will be administered via a nasogastric tube after induction of general anesthesia before embolization. |
| **Target number of research participants** | 484 patients (242 in each group) |
| **Study period** | Study duration: 4.25 years (jRCT publication date - March 31, 2026)  Case registration period: 3 years (January 1, 2022 - December 31, 2024)  Inclusion, implementation, and observation period: 3.25 (January 1, 2022 - March 31, 2025) |
| **Number of research facilities** | 44 Facilities  Department of Endovascular surgery, Tokyo Medical and Dental University, Tokyo, Japan; Department of Neurosurgery, National Hospital Organization Disaster Medical Center, Tokyo, Japan; Department of Neurosurgery, Japanese Red Cross Musashino Hospital, Tokyo, Japan; Department of Neurosurgery, Tsuchiura Kyodo General Hospital, Ibaraki, Japan; Department of Neurosurgery, Ome Medical Center, Tokyo, Japan; Department of Neurosurgery, Soka Municipal Hospital, Saitama, Japan; Department of Neurosurgery, Tokyo Metropolitan Toshima Hospital, Tokyo, Japan; Department of Neurosurgery, JA Toride Medical Center, Ibaraki, Japan; Department of Neurosurgery, Fujiyoshida Municipal Hospital, Yamanashi, Japan; Department of Neurosurgery, Shuuwa General Hospital, Saitama, Japan; Department of Neurosurgery, Tokyo Bay Urayau Ichikawa Medical Center, Chiba, Japan; Department of Neurosurgery, Shioda Memorial Hospital,Chiba,Japan; Department of Neurosurgery, Tokyo Kita Medical Center, Tokyo, Japan; Department of Neuroendovascular surgery, Jichi Medical University Saitama Medical Center, Saitama, Japan; Department of Neurosurgery, Kanto Rosai Hospital, Kanagawa, Japan; Department of Neurosurgery, Seisuikai Kajikawa Hospital, Hiroshima, Japan; Department of Neurosurgery, Higashiyamato Hospital, Tokyo, Japan; Department of Neuroendovascular surgery, Tokyo Metropolitan Police Hospital, Tokyo, Japan; Department of Neurosurgery, Gifu University Graduate School of Medicine, Gifu, Japan; Department of Neurosurgery, Center Hospital of the National Center for Global Health and Medicine, Tokyo, Japan; Department of Neurosurgery, Asahi General Hospital, Chiba, Japan; Division of Neurosurgery, Department of Brain and Neurosciences, Faculty of Medicine, Tottori University; Department of Neurosurgery, Tokushima University Hospital, Tokushima, Japan; Department of Neurosurgery, Graduate School of Medical Sciences, Kyushu University, Fukuoka, Japan; Department of Neurosurgery, Kanazawa University Hospital, Kanazawa, Japan; Department of Neurosurgery, Nagoya Tokushukai General Hospital, Aichi. Japan; Department of Neurosurgery, Graduate School of Medical Science, Kyoto Prefectural University of Medicine, Kyoto, Japan; Department of Neurosurgery, Saga Prefectural Hospital Koseikan, Saga, Japan; Department of Neurosurgery, Japanese Red Cross Society Kyoto Daini Hospital, Kyoto, Japan; Department of Neurosurgical, Seisho Hospital, Kanagawa, Japan; Department of Neurosurgery, Shinshu University Hospital, Nagano, Japan; Department of Neurosurgery, Hyogo Medical University Hospital, Hyogo, Japan; Department of Neurosurgery, Kyoto Saiseikai Hospital, Kyoto, Japan; Department of Endovascular Neurosurgery, Saitama Medical University International Medical Center, Saitama, Japan; Department of Neuroendovascular surgery, National Hospital Organization Kyushu Medical Center, Fukuoka, Japan; Department of Neurosurgery, Chiba Emergency and Psychiatric Medical center, Chiba, Japan; Department of Neurosurgery, Fukuoka University Chikushi Hospital, Fukuoka, Japan; Department of Neurosurgery, Kurume University Hospital, Fukuoka, Japan; Department of Neurosurgery, Yokohama Shintoshi Neurosurgical Hospital, Kanagawa, Japan; Department of Neurosurgery, Kimitsu Chuo Hospital, Chiba, Japan; Department of Neurosurgery, Dokkyo Medical University Saitama Medical Center, Saitama, Japan; Department of Neurosurgery, Tokyo Metropolitan Bokutoh Hospital, Tokyo, Japan; Department of Neurosurgery, Saitama Sekishinkai Hospital, Saitama, Japan; Department of Neurosurgery, Kyorin University Hospital, Tokyo, Japan |
| **Reseach implementation structure** | Principal investigator: Professor Kazutaka Sumita, Department of Endovascular, Tokyo Medical and Dental University, Tokyo, Japan, [sumita.nsrg@tmd.ac.jp](mailto:sumita.nsrg@tmd.ac.jp)  Local chief investigator:  Kazutaka Sumita, Department of Endovascular, Tokyo Medical and Dental University, Tokyo, Japan, sumita.nsrg@tmd.ac.jp  Keigo Shigeta, Department of Neurosurgery, National Hospital Organization Disaster Medical Center, Tokyo, Japan, [shigetak5@yahoo.co.jp](mailto:shigetak5@yahoo.co.jp),  Yohei Sato, Department of Neurosurgery, Japanese Musashino Red Cross Hospital, Tokyo, Japan, sato@sb4.so-net.ne.jp  Shin Hirota, Department of Neurosurgery, Tsuchiura Kyodo General Hospital, Tokyo, Japan, [hiroshin_21@yahoo.co.jp](mailto:hiroshin_21@yahoo.co.jp)  Jun Karakama, Department of Neurosurgery, Ome medical center, Tokyo, Japan, [sofas_float@yahoo.co.jp](mailto:sofas_float@yahoo.co.jp)  Yoshikazu Yoshino, Department of Neuroendovascular surgery, Jichi Medical University Saitama Medical Center, Saitama, Japan, [yoshino.evs@jichi.ac.jp](mailto:yoshino.evs@jichi.ac.jp)  Yosuke Ishi, Department of Neurosurgery, Kanto Rosai Hospital, Kanagawa, Japan, [yishii0712@icloud.com](mailto:yishii0712@icloud.com)  Mutusya Hara, Department of Neurosurgery, Tokyo Metropolitan Toshima Hospital, Tokyo,  Japan, [mutsuya_hara@tmhp.jp](mailto:mutsuya_hara@tmhp.jp)  Toshihiro Yamamura, Department of Neurosurgery, JA Toride Medical Center, Ibaraki, Japan, [yama.toshi1216@gmail.com](mailto:yama.toshi1216@gmail.com)  Motoshigeru Yamashina, Department of Neurosurgery, Soka Municipal Hospital, Saitama, Japan, [yshina132@yahoo.co.jp](mailto:yshina132@yahoo.co.jp)  Shogo Imae, Department of Neurosurgery, Fujiyoshida Municipal Hospital, Yamanashi, Japan, imae.nsrg@tmd.ac.jp  Kana Sawada, Department of Neurosurgery, Tokyo Bay Urayasu Ichikawa Medical Center, Tokyo, Japan, kanasa@jadecom.jp  Yoshiki Obata, Department of Neurosurgery, Tokyo Kita Medical Center, Tokyo, Japan, obata-tmd@umin.ac.jp  Tadahiro Ishiwada, Department of Neurosurgery, Shioda Memorial Hospital, Chiba, Japan, tadahiro0296@yahoo.co.jp  Naoki Taira, Department of Neurosurgery, Shuuwa General Hospital, Saitama, Japan, taira-md@shuuwa-gh.or.jp  Tatsuya Mizoue, Department of Neurosurgery, Suiseikai Kajikami Hospital, Hiroshima, Japan, [mizoue@suiseikai.jp](mailto:mizoue@suiseikai.jp)  Masahiro Indo, Department of Neurosurgery, Higashiyamato Hospital, Tokyo, Japan, clea_danes@yahoo.co.jp  Hiroaki Sato, Department of Neuroendovascular surgery, Tokyo Metropolitan Police Hospital, Tokyo, Japan, [hirosatou-nsu@nifty.com](mailto:hirosatou-nsu@nifty.com)  Yukiko Enomoto, Department of Neurosurgery, Gifu University Graduate School of Medicine, Gifu, Japan, enomoto.yukiko.k1@f.gifu-u.ac.jp  Masato Inoue, Department of Neurosurgery, Center Hospital of the National Center for Global Health and Medicine, Tokyo, Japan, [inmasato@hosp.ncgm.go.jp](mailto:inmasato@hosp.ncgm.go.jp)  Hidetoshi Mochida, Department of Neurosurgery, Asahi General Hospital, Chiba, Japan, [motty-hi@js8.so-net.ne.jp](mailto:motty-hi@js8.so-net.ne.jp)  Makoto Sakamoto, Division of Neurosurgery, Department of Brain and Neurosciences, Faculty of Medicine, Tottori University,Tottori, Japan, sakamako@tottori-u.ac.jp  Yasushi Takagi, Department of Neurosurgery, Tokushima University Hospital, Tokushima, Japan, ytakagi@tokushima-u.ac.jp  Koichi Arimura, Department of Neurosurgery, Graduate School of Medical Sciences, Kyushu University, Fukuoka, Japan, [arimura.koichi.001@m.kyushu-u.ac.jp](mailto:arimura.koichi.001@m.kyushu-u.ac.jp)  Koichi Misaki, Department of Neurosurgery, Kanazawa University Hospital, Kanazawa, Japan, [misaki@med.kanazawa-u.ac.jp](mailto:misaki@med.kanazawa-u.ac.jp)  Takayuki Amamo, Department of Neurosurgery, Nagoya Tokushukai General Hospital, Aichi, Japan, [ama37tk@gmail.com](mailto:ama37tk@gmail.com)  Masataka Nanto, Department of Neurosurgery, Graduate School of Medical Science, Kyoto Prefectural University of Medicine, Kyoto, Japan, [nanto@koto.kpu-m.ac.jp](mailto:nanto@koto.kpu-m.ac.jp)  Kenichi Matsumoto, Department of Neurosurgery, Saga Prefectural hospital Koseikan, Saga, Japan, [matsumoto-k@koseikan.jp](mailto:matsumoto-k@koseikan.jp)  Nobukuni Murakami, Department of Neurosurgery, Japanese Red Cross Society Kyoto Daini Hospital, Kyoto, Japan, nobukuni.mrkm@gmail.com  Masataka Takeuchi, Department of Neurosurgical, Seisho Hospital, Kanagawa, Japan, [masatakatakeuchi@hotmail.com](mailto:masatakatakeuchi@hotmail.com)  Yoshiki Hanaoka, Department of Neurosurgery, Shinshu University Hospital, Nagano, Japan, [hanaoka@shinshu-u.ac.jp](mailto:hanaoka@shinshu-u.ac.jp)  Shinichi Yoshimura, Department of Neurosurgery, Hyogo Medical University Hospital, Hyogo, Japan, s-yoshi@hyo-med.ac.jp  Junichi Miyamoto, Department of Neurosurgery, Kyoto Saiseikai Hospital, Kyoto, Japan, [miyapoo850@yahoo.co.jp](mailto:miyapoo850@yahoo.co.jp)  Shinya Kohyama, Department of Endovascular Neurosurgery, Saitama Medical University International Medical Center, Saitama, Japan, [sk3821@5931.saitama-med.ac.jp](mailto:sk3821@5931.saitama-med.ac.jp)  So Tokunaga, Department of Neuroendovascular surgery, National Hospital Organization Kyushu Medical Center, Fukuoka, Japan, tokunaga_so@yahoo.co.jp  Toshihiro Yamauchi, Department of Neurosurgery, Chiba Emergency and Psychiatric Medical center, Chiba, Japan, [toshihiro.yamauchi@chiba-emc.jp](mailto:toshihiro.yamauchi@chiba-emc.jp)  Toshio Higashi, Department of Neurosurgery, Fukuoka University Chikushi Hospital, Fukuoka, Japan, toshio.higashi@gmail.com  Masaru Hirohata, Department of Neurosurgery, Kurume University Hospital, Fukuoka, Japan, [hiroha@kurume-u.ac.jp](mailto:hiroha@kurume-u.ac.jp)  Masafumi Morimoto, Department of Neurosurgery, Yokohama Shintoshi Neurosurgical Hospital, Kanagawa, Japan, gnsmasafumi@gmail.com  Hayasaka Michihiro, Department of Neurosurgery, Kimitsu Chuo Hospital, Chiba, Japan, mmmsy@yb3.so-net.ne.jp  Tomoji Takigawa, Department of Neurosurgery, Dokkyo Medical University Saitama Medical Center, Saitama, Japan, [takigawa@dokkyomed.ac.jp](mailto:takigawa@dokkyomed.ac.jp)  Kazuo Hanakawa, Department of Neurosurgery, Tokyo Metropolitan Bokutoh Hospital, Tokyo, Japan, kazuo_hanakawa@tmhp.jp  Yukihiro Hidaka, Department of Neurosurgery, Saitama Sekishinkai Hospital, Saitama, Japan, [yuki.hidaka0426@gmail.com](mailto:yuki.hidaka0426@gmail.com)  Hirofumi Nakatomi, Department of Neurosurgery, Kyorin University Hospital, Tokyo, Japan, [hirofuminakatomi@gmail.com](mailto:hirofuminakatomi@gmail.com)  Study manager: Ishiguro Megumi, Health Science R&D Centre, Tokyo Medical and Dental University, Tokyo, Japan, ishiguro.srg2@tmd.ac.jp  Data manager: Pariko Yorozu, Health Science R&D Centre, Tokyo Medical and Dental University, Tokyo, Japan, [p.yorozu.pth1@tmd.ac.jp](mailto:p.yorozu.pth1@tmd.ac.jp)  Monitoring Director: Makoto Ishii, Health Science R&D Centre, Tokyo Medical and Dental University, Tokyo, Japan, [ishii.m.adm@tmd.ac.jp](mailto:ishii.m.adm@tmd.ac.jp)  Coordination Secretariat: Emi Yoshida, Health Science R&D Centre, Tokyo Medical and Dental University, Tokyo, Japan, e-yoshida.adm@tmd.ac.jp  Statistical Analysis Director: Akihiro Hirakawa, Department of Clinical Biostatistics, Graduate School of Medical and Dental Sciences, Tokyo Medical and Dental University, Tokyo, Japan,  [a-hirakawa.crc@tmd.ac.jp](mailto:a-hirakawa.crc@tmd.ac.jp)  Statistical analysts :  Hiroyuki Sato, Department of Clinical Biostatistics, Graduate School of Medical and Dental Sciences, Tokyo Medical and Dental University, Tokyo, Japan, h-sato.crc@tmd.ac.jp  Ryoichi Hanazawa, Department of Clinical Biostatistics, Graduate School of Medical and Dental Sciences, Tokyo Medical and Dental University, Tokyo, Japan, r-hanazawa.crc@tmd.ac.jp  Sasaki Seiji, Department of Clinical Biostatistics, Graduate School of Medical and Dental Sciences, Tokyo Medical and Dental University, Tokyo, Japan, m-sasaki.crc@tmd.ac.jp  Independent Review Board (IRC) (Central Imaging Assessment Committee):  Jun Ooyama, Department of Radiology, Tokyo Medical and Dental University, Tokyo, Japan,  ooyamarad@tmd.ac.jp  Kota Yokoyama, Department of Radiology, Tokyo Medical and Dental University, Tokyo, Japan, kota1986ky@yahoo.co.jp  Shoko Hara, Department of Neurosurgery, Tokyo Medical and Dental University, Tokyo, Japan, shara.nsrg@tmd.ac.jp[(shara.nsrg@tmd.ac.jp)](mailto:shara.nsrg@tmd.ac.jp) |
| **Management of Samples and Information** | The sample and information shall be stored for 10 years in a key archive of the Department of Endovascular Surgery by Kazutaka Sumita, and then dissolved and discarded so that personal information cannot be identified. |
| **Ethical guidelines** | All persons involved in the study follow the spirit of the Declaration of Helsinki of the World Medical Association (revised October 2013), as well as the Clinical Research Act (Act No. 16 of 2017) and the Enforcement Regulations of the Clinical Research Act. |
| **Certified Clinical Research Review Board** | Before conducting this study, the Certified Clinical Research Review Board will review the ethical, scientific, and medical relevance of this study. This study will be conducted after being registered with jRCT after obtaining approval from the Certified Clinical Research Review Board and with permission from the administrators of the participating medical institutions. If the results of deliberation by the Certified Clinical Research Review Board are "continued," the Study Protocol or Case Report Form (CRF), informed consent form, etc. will be revised based on the results of the deliberation, approved, and the above procedure will be carried out, followed by the present study. In addition, the Certified Clinical Research Review Board continuously reviews whether the study has been properly conducted at least once a year. |
| **Research points**  **Summary** | ・Collection of human specimens □ Yes ■ No  ・Monitoring ■ Yes □ No  (Monitoring: Health Science R&D Centre, Tokyo Medical and Dental University, Tokyo, Japan)  ・Audit □ Yes ■ No  ・Invasion □ Yes ■ With slight invasion □ No  ・Multicenter collaborative research □Tokyo Medical and Dental University only ■ Multicenter principal □ Multicenter sharing  ・Samples and information received from other facilities ■ Yes □ No  ・Provision of samples and information overseas □ Yes ■ No  ・Location of Samples, Information, Records, etc.: Department of Endovascular surgery office  ・Storage manager : Kazutaka Sumita  ・Storage period: 10 years  ・Discard method: When information needs to be discarded, eliminate the data from the stored computer, treat the output of the print, etc. with a shredder, etc. to discard it.  ・Secondary availability ■ Yes □ No  ・Storage of records of provision  ■ Described in this document □ Described in attached sheet □ MTA □ Not applicable  ・Rewards  □ Yes ■ None  ・Anonymization method: Corresponding table  ・Compensation insurance　 ■ Yes □ No  ・Research fund: ■ Operating expenses □ Grants-in-Aid for Scientific Research ■ Subsidies other than Grants-in-Aid for Scientific Research ■ Donations □ Contract and collaborative research |

#

# **1.** **Study Background**

Endovascular treatment (EVT) has become an established treatment for cerebral aneurysms ^1, 2^. However, EVT has a potential risk of thromboembolic complications because it involves inserting a foreign body into the body ^3, 4^. Dual antiplatelet therapy with aspirin and clopidogrel is a common EVT for unruptured cerebral aneurysms that prevents thrombotic complications ^5^. This dual antiplatelet therapy has enabled the use of useful devices such as stenting and balloons, which are support devices for coil embolization or flow diverter placement, as well as the treatment of complex forms of aneurysms with a high degree of difficulty that has been previously difficult to treat ^6-8^.

EVT for ruptured cerebral aneurysms is associated with a high incidence of intraoperative thromboembolism because of hypercoagulable state as a bioprotective response to subarachnoid hemorrhage (SAH) ^9, 10^. Cognard et al. reported complications in 13.3% of cases and severe neurological sequelae or death in 4.2%^11^. Therefore, controlling thromboembolic complications is important in the EVT of ruptured cerebral aneurysms.

In 2006, Rie et al. reported that the administration of antiplatelet agents during EVT for ruptured cerebral aneurysms reduces thromboembolic morbidity^12^. However, in 2009, a subanalysis of the International Subarachnoid Aneurysm Trial (ISAT), a randomized controlled trial (RCT) of craniotomy versus EVT for ruptured cerebral aneurysms, reported that intraoperative or postoperative antiplatelet use in EVT for ruptured cerebral aneurysms did not improve SAH outcomes. The biggest problem with this study was that the antiplatelet agents were administered either intraoperatively or postoperatively, meanwhile, they should have been administered preoperatively as antiplatelet agents to prevent thrombus formation during treatment. Recently, the usefulness of preoperative administration of antiplatelet agents to prevent thromboembolism complications in EVT in the acute stage for ruptured cerebral aneurysms has been reported ^6, 14^. Increased bleeding complications are a concern when administering antiplatelet agents, which may promote bleeding in the acute phase of a ruptured cerebral aneurysm. In addition to the risk of aneurysmal rebleeding, those with ruptured cerebral aneurysms have a potentially intrinsic risk of hemorrhagic complications, including the risk of gastrointestinal bleeding resulting from increased intracranial pressure associated with SAH; the risk of hemorrhagic complications during additional surgical treatments such as ventricular or spinal drainage, which may be required if acute hydrocephalus is complicated; and the risk of increased intracerebral hematoma. However, in previous retrospective studies on preoperative and intraoperative antiplatelet administration in EVT for ruptured cerebral aneurysms, hemorrhagic morbidity has not increased, and the safety of its use^6, 12^.

Thus, although the usefulness and safety of preoperative antiplatelet therapy for the EVT of ruptured cerebral aneurysms in the acute phase have been reported, they have not been tested in an RCT. Therefore, in this study, among patients scheduled for EVT during the acute phase (within 72 h of onset) for ruptured cerebral aneurysms, consented patients will be randomly assigned double-blindly to receive aspirin 200 mg via a nasogastric tube or placebo after induction of general anesthesia before EVT. To evaluate the occurrence of intraoperative thromboembolic complications and the presence or absence of concordant neurological dysfunction on MRI DWI taken within 6-48 h after surgery, global cerebral ischemic events within 14 days after MRI, all hemorrhagic events within 14 days of enrollment, and mRS at 3 months. This study aimed to determine the safety and efficacy of preoperative aspirin administration in the EVT of acute-phase ruptured cerebral aneurysms.

# **2.** **Purpose of the study**

　During EVT of ruptured cerebral aneurysms, thromboembolic complications are known to be uncontrollable therapeutic complications and further improvement in treatment outcomes can be achieved by controlling these complications. We hypothesized that the administration of preoperative antiplatelet agents for EVT may control thromboembolic complications.

　For embolization of acutely ruptured saccular cerebral aneurysms, patients will be randomized to receive aspirin or placebo preoperatively to test for differences in the incidence rate of intraoperative thromboembolic events, new postoperative neurological dysfunctions, new cerebral infarctions on postoperative MRI, perioperative cerebral ischemic events, any hemorrhagic events, and 3-month mRS between the two groups.

# **3.** **Summary of Drugs Used in Clinical Research**

When performing embolization of a ruptured cerebral aneurysm, a nasogastric tube will be inserted after the induction of general anesthesia, and 200 mg of aspirin powder or 200 mg of placebo-lactose will be dissolved in approximately 20mL of tepid water via a nasogastric tube.

・Aspirin (Aspirin)

Date of approval: August 6, 1985

　Approval Number: 16000AMZ03805000

　The products were manufactured and marketed by Mylan Pharmaceutical Co. Ltd.

　Sales: Pfizer Pharmaceuticals Inc.

　Dosage form: Powder (crystals)

　Storage: Storage at room temperature

・Lactose (Lactose Hydrate)

Manufacturing approval Date: February 1, 2008

Approval Number: 27A2X00206

Manufactured and marketed by Mylan Pharmaceutical Co., Ltd.

　Sales: Pfizer Pharmaceuticals Inc.

Dosage form: Powder (crystals)

　Storage: Storage at room temperature

# **4. Patient population**

## **4-1.** **Inclusion criteria**

**The study will be conducted on patients who meet all of the following conditions.**

1. Patients with ruptured cerebral aneurysms in the acute phase
2. Patients scheduled for aneurysm embolization within 72 h of onset
3. Patients aged 20 years or older at the time of presentation
4. Patients for whom written informed consent will be obtained from the participants or their legally acceptable representatives (e.g., spouse, parent, or adult child) for participation in the study.

[Rationale] 1), 2), 3) Basic target diseases and appropriate timing of treatment.

4) Consent from the person is desirable, but owing to the nature of the disease, which is often accompanied by a disturbance of consciousness, consent from a legally acceptable person qualifies the participant.

## **4-2. Exclusion criteria**

**Exclude patients who meet any of the following criteria**

1) Individuals without independent premorbid activities of daily living (pre-stroke mRS ≧4).

2) Patients with dissecting cerebral aneurysms in whom parent artery occlusion was attempted

3) Patients with recurrent ruptured cerebral aneurysms after cerebral aneurysm coil embolization

4) Patients with cerebral arteriovenous malformations, moyamoya disease, and other cerebral aneurysms or ruptured infectious cerebral aneurysms

5) Patients treated simultaneously for two or more aneurysms

6) Patients already taking antiplatelet drugs

7) Patients with a history of allergy to lactose, aspirin, or salicylic acid preparations

8) Patients who cannot undergo MRI

9) Patients with contraindications to aspirin

10) Other patients considered by the treating physician not appropriate to participate in the study

[Rationale Setting] 1), 2), 3), 4), 5), 6) Effect on Evaluation of Efficacy

　　　　　　　　 　7), 8), 9), 10) For safety considerations

# **5.** **Method of obtaining consent from the patients**

Before the study, the investigator or sub-investigator in each collaborative study period (each participating institution) will provide the patients scheduled for EVT for a ruptured cerebral aneurysm and their legally acceptable representative with an easy-to-understand explanation of the study. Using the latest informed consent form approved by the Certified Clinical Research Review Board preoperatively, written informed consent to participate in the study will be obtained. When obtaining consent, sufficient time and opportunity to ask questions about participating in the study will be provided. All questions will be sufficiently answered. The principal investigator or physician responsible for the explanation will confirm whether the subject or his/her legally acceptable representative fully understands the content of the information and agrees with it. The investigator or sub-investigator shall enter the date of explanation on the informed consent form and sign the form with or without a seal. The participant or his/her representative shall fully understand the contents of the written information, agree with the date of consent, and affix his/her name and seal or signature to the written agreement.

The investigator or sub-investigator shall supply the participant or his/her legally acceptable representative with a copy of the signed and sealed consent form and retain the original consent form at the relevant medical institution.

In the event of a decision to continue participating in the study, the investigator or sub-investigator shall revise the explanatory documents and re-explain to the participant using the revised explanatory documents. Consent for the continuation of the study will be obtained from the participants or the legally acceptable representatives of their free will.

When a participant or a legally acceptable representative offers withdrawal of consent, this shall be noted in the medical records. The participant or the legally acceptable representative will sign the consent withdrawal form, and the principal investigator or sub-investigator will sign the date of confirmation and name and seal. The investigator or sub-investigator shall supply the participants or their proxies with a copy of the signed and sealed consent withdrawal form, and the original consent withdrawal form will be retained at the relevant medical institution.

# **6.** **Methods of Research (Content)**

This will be a double-blind, parallel-group study to test the efficacy and safety of aspirin in patients with ruptured cerebral aneurysms who will be randomly assigned to receive aspirin or a placebo before EVT. The following diagram describes the procedure of this study, the decision of the diagnosis and treatment plan of the ruptured cerebral aneurysm case recommended in this study, a technique of EVT, correspondence in the thrombosis generation during the operation, and postoperative management.

**Schema of the study**

**
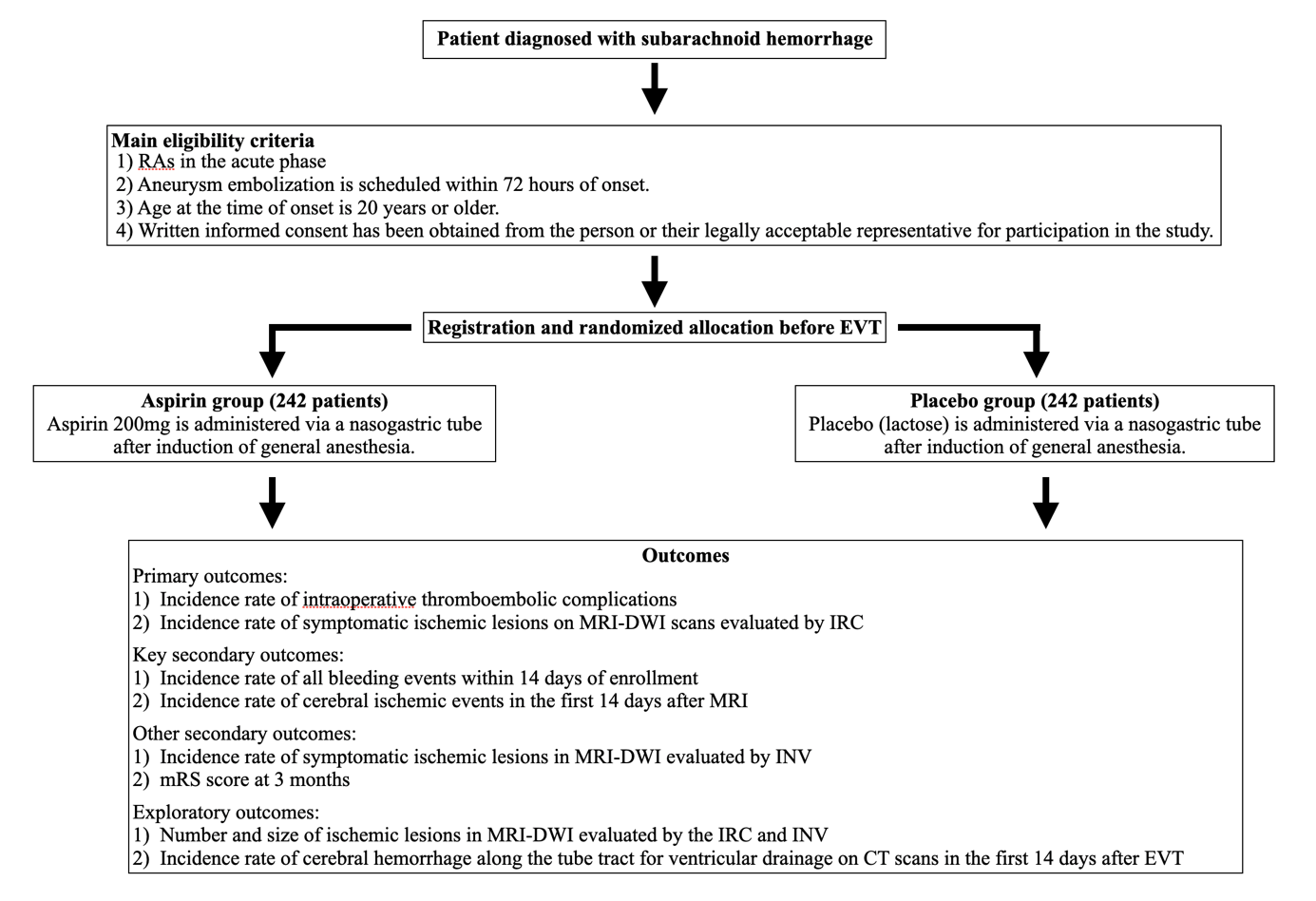
**

**Diagnosis and treatment decision of ruptured cerebral aneurysm.**

　After diagnosing subarachnoid hemorrhage by head CT/MRI, the site and configuration of the ruptured cerebral aneurysm are evaluated by computed tomography angiography/magnetic resonance angiography to identify the ruptured site. The patient's condition, including neurologic findings, vital signs, hematologic tests, chest CT and radiography, and electrocardiographic findings at the time of the visit, will be assessed, and the treatment plan will be decided in the department. If EVT is planned, it will be considered that the patient meets the inclusion criteria and does not meet the exclusion criteria. If consent is obtained from the patient or a relative, the patient will be enrolled and assigned. Patients will be provided systemic management, including sedation and antihypertensive agents, and will await surgery. Preoperative systemic management, including antihypertensive and sedative methods, and management of hydrocephalus (whether CSF drainage is performed preoperatively or postoperatively) are left to each institution.

**Endovascular treatment technique and intraoperative response to thrombus.**

　Cerebral aneurysm embolization with EVT within 72 h of presentation. EVT is performed under general anesthesia. Administration of a specified number of drugs (aspirin 200 mg or lactose 200 mg) at the time of enrollment/allocation will be performed by the physician in charge of anesthesia or the study sub-investigator via the gastric tube after induction of general anesthesia. The surgeon group will not be administered any visible medication. A cerebral aneurysm embolization will be performed.

Cerebral aneurysm embolization is performed via the transfemoral, radial, brachial, and carotid arteries and a guiding catheter is placed in the internal or vertebral carotid artery. Intraoperatively, heparin, an anticoagulant, is administered intravenously at the discretion of the surgeon, and systemic heparinization is performed to activate coagulation time (ACT) from 250 s to 300 s. A microcatheter and microguidewire are used to place the microcatheter in the aneurysm. A coil for aneurysm embolization is placed in the aneurysm via a microcatheter placed in the aneurysm. If needed, a distal access catheter that allows stable distal vascular access, balloon catheter neck modeling, and double-catheter embolization with two microcatheters may be used. Treatment is terminated when sufficient embolization is achieved to prevent aneurysm re-rupture.

Intraoperatively, if there is evidence of cerebral angiographically evident thrombosis around the aneurysmal neck or in the parent artery (primary endpoint 1), ACT will be measured again immediately, and heparinization will be intensified to provide an ACT > 300 s, or Ozagrel Sodium 80 mg administered intravenously. To confirm the resolution of the thrombus, repeat angiography will be performed as appropriate. If there is a tendency to exacerbate the thrombus, endovascular procedures such as thrombus disruption with a balloon catheter and thrombus retrieval with a stent retriever or thrombus aspiration catheter should be considered. When required, the emergency key of the case will be opened to check the details of the drug administered promptly; if the drug administered is aspirin powder 200 mg, clopidogrel 300 mg will be administered, or if the drug administered is lactose 200 mg, aspirin powder 200 mg and clopidogrel 300 mg will be administered via a nasogastric tube.

　If stent placement is judged to be required due to significant deviation of the coils into the parent artery during the operation, the emergency room should be opened and the medication checked. Clopidogrel 300 mg (or aspirin 200 mg and clopidogrel 300 mg) should be administered via a nasogastric tube to load the antiplatelet agent, and intracranial stent placement should be performed.

**Standard postoperative management**

MRI will be performed within 6–48 h after surgery, and assessment of ischemic lesions and concordant neurological dysfunction will be performed by the investigator and sub-investigator (INV), as well as assessment by IRC separately, with the outcome of IRC as the primary endpoint (primary endpoint 2). Assessment by IRC is based on CT simplex or MRI (CTA or MRA of the head before surgery, plain CT of the head within 48 h after surgery, clinical information on the cerebral aneurysm at the time of surgery (prior treatment of the aneurysm, location, surgical intervention before the treatment of the aneurysm), and worsening of surgical intervention and neurological outcome after the treatment of the cerebral aneurysm between the time of surgery and MRI imaging.

Postoperatively, the patient is managed systemically with standard-of-care (continuous intravenous infusion of nicardipine hydrochloride 120-600μg/kg/Hr, intravenous fasudil hydrochloride hydrate 90 mg/day, ozagrel sodium 80 mg/day, etc.) for cerebral vasospasm discussed at the participating institutions. However, the dose may change at the discretion of each institution. If perioperative surgical intervention, including cerebrospinal fluid drainage or EVT for symptomatic cerebral vasospasm (percutaneous cerebral angioplasty or fasudil hydrochloride hydrate arterial infusion therapy), is required, it should be performed at the discretion of the individual institution. The basic protocol requires a head CT performed within 48 h after surgery and 14 ± 2 days after surgery. Imaging will be performed as appropriate during this period, and all cerebral ischemic events within 14 days after MRI and all hemorrhagic events within 14 days after enrollment will be evaluated. In addition, the mRS score 3 months after the onset of subarachnoid hemorrhage will be assessed by the investigator and sub-investigator in the outpatient clinic on a face-to-face basis. If the patient cannot visit the hospital or is hospitalized in another hospital, the patient will be evaluated via telephone interviews.

## **6-1.** **Type and design of studies**

　This will be a randomized, placebo-controlled, double-blind, parallel-group study.

## **6-2.** **Study period**

Period of study: 4.25 years (jRC announcement date: March 31, 2026)

　 Case registration period: 3 years (January 1, 2022 - December 31, 2024)

　 Inclusion, implementation, and observation period: 3.25 years (January 1, 2022 - March 31, 2025)

## **6-3.** **Dosage and administration and duration of administration of the research drug**

After obtaining and assigning informed consent, when performing aneurysm embolization for a ruptured cerebral aneurysm, a nasogastric tube is inserted after induction of general anesthesia, and aspirin-powder 200mg or placebo-lactose 200mg is dissolved in tepid-water 20mL via a nasogastric tube.

・Aspirin (Aspirin)

Date of approval: August 6, 1985

　Approval Number: 16000AMZ03805000

　Manufactured and marketed by Mylan Pharmaceutical Co., Ltd.

　Sales: Pfizer Pharmaceuticals Inc.

　Dosage form: powder (crystals)

　Storage: Storage at room temperature

・Lactose (Lactose Hydrate)

Manufacturing approval Date: February 1, 2008

Approval Number: 27A2X00206

Manufactured and marketed by Mylan Pharmaceutical Co., Ltd.

　Sales: Pfizer Pharmaceuticals Inc.

Dosage form: powder (crystals)

　Storage: Storage at room temperature

## **6-4.** **Patient registration and allocation method**

<Methods of case registration>

The investigator or sub-investigator,

1. Written consent shall be obtained from the patient or proxy consenter.
2. Interactive Web Response Systems(IWRS) enter the inclusion/exclusion criteria, and allocation adjustment factors, and receive the drug number.
3. Receive the study drugs (two packages including the reserve) for the drug number. Test drugs are anesthetic

Hand it to your doctor. Properly manage the unused reserve after surgery and at a later date.

dispose of the drug at each facility when it expires.

<Methods of Randomization>

Each center will be allocated 1:1 to aspirin-and placebo-treated groups by dynamic allocation (minimization method) with age (≧65 years, <65 years), sex (male, female), World Federation of Neurosurgical Societies (WFNS) grade (I-III, IV-V), and modified Fisher grade (0-2, 3-4 as allocation-adjusted factors.

<How to blind study drugs>

In this study, a double-blind method will be employed, and the study personnel and participants, other than the pharmacist preparing the study drug and allocation manager will be blinded.

1. Confirm the indistinguishability of the test drug.
2. A corresponding table will be prepared consisting of the allocation group (aspirin or placebo) and the drug number.
3. The drug number is assigned to the test drug based on the corresponding table.
4. Provide the correspondence table to the allocation system administrator.
5. The corresponding table should be stored until unblinding and should not be disclosed to other persons. Unblinding is not to be performed until the test is completed, and the collection or exclusion of each participant is fixed, except during an emergency.

To ensure the quality of the test drug, the procedure for preparation of the test drug and blinding is carried out approximately every 3 months.

<Opening the emergency key>

When the principal investigator or sub-investigator judges that the occurrence of a serious adverse event for which a causal relationship to the investigational drug cannot be ruled out or the appearance of thrombus formation evident on cerebral angiography around the aneurysm or in the parent artery during surgery requires the opening of an emergency key to ensure the safety of the participant, the emergency key will be opened in the allocation system to check the allocation of the investigational drug in the relevant participant and take appropriate action. If an emergency key is opened, the principal investigator or sub-investigator records the reason for disclosure and immediately contacts the test office.

## 6-5. Responses after study completion

Continue treatment in routine insurance practice after completion of the study

# **7.** **Endpoint**

## **7-1.** **Items related to the evaluation of efficacy**

　Primary outcomes

1) Incidence rate of intraoperative thrombotic complications

It is defined as the proportion of patients with evidence of cerebral angiographically evident thrombus formation in the vicinity of the aneurysm neck or parent artery during surgery.

2) Incidence rate of symptomatic ischemic lesions on MRI diffusion-weighted imaging (DWI) evaluated by the Independent Review Committee (IRC)

It is defined as the percentage of patients with acute-phase ischemic lesions on MRI-DWI performed 6–48 h postoperatively and with concordant neurologic findings.

Key secondary outcomes

1) Incidence rate of all bleeding events within 14 days of enrollment

As a basic protocol, a head CT is performed within 48 h postoperatively and at 14±2 days postoperatively. Additionally, imaging evaluations were conducted as required. The incidence rate of all bleeding events is defined as the percentage of patients with a ruptured aneurysm (symptomatic subarachnoid or intracerebral hemorrhage due to rupture of an aneurysm of interest), a hemorrhagic stroke (symptomatic subarachnoid and intracerebral hemorrhage not due to rupture of target aneurysm, or the proportion of patients who experienced "major hemorrhage" according to ISTH criteria within 14 days post-registration.

2) Incidence rate of cerebral ischemic events in the first 14 days after MRI

The basic protocol is head CT performed within 6–48 h after surgery and 14 ± 2 days after surgery. This is defined as the percentage of patients with transient ischemic attack within 14 ± 2 days after MRI (neurological symptoms lasting less than 24 h with or without imaging findings) or ischemic stroke (focal neurological symptoms lasting more than 24 h and ischemic lesions confirmed by head CT or MRI) during the study period.

Other secondary outcomes

1) Incidence rate of symptomatic ischemic lesions in MRI-DWI evaluated by INV.

2) mRS at 3 months

Assessment is based on mRS scoring 3 months after the onset of subarachnoid hemorrhage.

　Exploratory outcomes

1) Ischemic lesions on MRI-DWI (number and size: 0–4 mm, 5–9 mm, 10–14 mm, 15 mm or greater, categorized as IRC or INV)

2) Incidence rate of cerebral hemorrhage along the tube tract for ventricular drainage on CT scans in the first 14 days after EVT.

## **7-2.** **Items related to safety evaluation**

**Evaluation of adverse events and diseases**

**Definition of adverse events**

An adverse event (AE) is any untoward medical occurrence in a participant receiving treatment and does not necessarily indicate a causal relationship with the study treatment. An adverse event can, therefore, be any unfavorable or unintended sign (including an abnormal laboratory finding), symptom, or disease occurring at the time of treatment, regardless of whether it is considered treatment-related. The case report form should include the name of the adverse event, date of onset, outcome, and causal relationship with the antiplatelet agents.

A serious adverse event (SAE) was defined as any of the following:

1) Death;
2) Life-threatening;
3) Those requiring hospitalization for treatment or prolongation of hospitalization;
4) Permanent or significant disability/dysfunction;
5) Congenital anomalies in offspring

**Definition of disease**

Adverse events suspected to be caused by the conduct of this study (diseases, disability, death, and infectious diseases, including abnormal laboratory values and symptoms) are referred to as diseases. A causal relationship with the study drug or procedure cannot be ruled out. Causality was determined by the investigator or sub-investigator.

Additionally, for diseases corresponding to serious adverse events, the procedures in 10-2 should be followed.

**Causality assessment**

The principal investigator or sub-investigator will determine whether there is a causal relationship between adverse events (death, occurrence of major hemorrhagic events, re-treatment of the target cerebral aneurysm, and re-bleeding of the target cerebral aneurysm) that occurred during this study and the causal relationship of this study.

1) Causality/causality cannot be ruled out: temporally related to study drug administration or procedures, reasonably judged to be due to the study drug or procedure, or unrelated to the study drug.

2) No causality: It can be judged as not being related based on the temporal relationship with the administration of the test drug or test procedure.

**Adverse event reporting**

Adverse events (death, occurrence of major hemorrhagic events, retreatment of the target cerebral aneurysm, and rebleeding of the target cerebral aneurysm) will be recorded in a case report form. Diseases, among other SAEs, are reported based on the procedures in 10-2.

**Expected adverse events**

　The expected adverse events in this study are as follows: refer to the package insert for updated information.

Side effects of aspirin

(1) Significant adverse reactions

1. Shock and anaphylaxis (incidence unknown): Shock or anaphylaxis (dyspnea, generalized flushing, angioedema, urticaria, etc.) may occur. Patients should be carefully monitored, and if any abnormalities are observed, administration should be discontinued and appropriate measures should be taken.

2. Intracranial hemorrhage such as cerebral hemorrhage,(initial symptoms: headache, nausea/vomiting, disturbed consciousness, hemiplegia, etc.) may occur. Patients should be carefully monitored, and if such symptoms are observed, administration should be discontinued and appropriate measures taken. Pulmonary, gastrointestinal, epistaxis and fundus hemorrhage may occur. Patients should be carefully monitored, and if such symptoms are observed, administration should be discontinued and appropriate measures taken.

3. Toxic epidermal necrolysis (TEN), oculomucocutaneous syndrome (Stevens-Johnson syndrome), and exfoliative dermatitis may also occur. Patients should be carefully monitored, and if such symptoms are observed, administration should be discontinued and appropriate measures should be taken.

4. Aplastic anemia, thrombocytopenia, and leukopenia: Patients should be carefully monitored, and if any abnormalities are observed, administration should be discontinued and appropriate measures should be taken.

5. Asthma attacks: It may induce asthmatic attacks.

6. In hepatic function disorders and jaundice with significant elevations in hepatic function disorder, AST (GOT), ALT (GPT and gamma-GTP) may occur. Patients should be carefully monitored, and if any abnormal findings are observed, appropriate measures, such as discontinuation of administration, should be taken.

7. Peptic ulcers, small and large intestine ulcers: Peptic ulcers such as gastric and duodenal ulcers with melena may occur. Small- and large-intestinal ulcers associated with gastrointestinal bleeding and perforation may also occur. Patients should be carefully monitored, and if any abnormalities are observed, the administration should be discontinued and appropriate measures should be taken.

(2) Since no domestic survey has been conducted to clarify the indications for adverse-reaction drugs and the frequency of adverse reactions in terms of administration and dosage, it is based on the frequency of adverse reactions caused by aspirin-based preparations used as antipyretic analgesics and anti-inflammatory agents.

|  | More than 5% or frequency unknown | 0.1~ <5% | <0.1% |
| --- | --- | --- | --- |
| Digestive system | Gastrointestinal disorders, vomiting, abdominal pain, heartburn, constipation, diarrhea, esophagitis, lip swelling, hematemesis, nausea | Nausea, anorexia, stomach discomfort |  |
| Hypersensitivity ^1)^ | Urticaria | Rash, edema |  |
| Hematologic ^2)^ |  |  | Anemia, decreased platelet function (prolonged bleeding time) |
| Skin | Pruritus, rash, wheal, sweating |  |  |
| Psychoneurological ^3)^ | Dizziness, agitation | Headache |  |
| Liver | Increased AST (GOT)  ALT (GPT) |  |  |
| Kidney |  |  | Kidney damage |
| Cardiovascular | Hypotension, vasculitis | Epigastric pain |  |
| Respiratory system | Bronchitis | Rhinitis |  |
| Sensory organ | Keratitis and conjunctivitis | Tinnitus, hearing loss |  |
| Miscellaneous ^4)^ | Hyperpnea, metabolic acidosis | Malaise | Hypoglycemia |

1. If any symptom occurs, the therapy should be discontinued.

2. If any abnormality is observed, osimertinib should be discontinued, and appropriate measures should be taken.

3. If any symptoms are observed, the dose should be reduced or administration should be discontinued.

4. Dose reduction or discontinuation (blood concentration may be markedly elevated).

In the foreign post-marketing surveillance of drugs used as antiplatelet agents, the number of patients with adverse reactions was 73 (87 adverse reactions) of the 2739 patients surveyed, and the incidence of adverse reactions was 2.67%. According to the classification of adverse drug reactions according to organs, gastrointestinal disorders (gastritis, gastrointestinal bleeding, ulcers, etc.) were the most common (2.81%), followed by hemorrhage/coagulopathy (hematoma, retinal hemorrhage, etc.) (0.15%), hypersensitivity (rash) (0.07%), psychiatric nervous system disorders (dizziness), and hematological disorders (anemia) were all 0.04%.

# **8.** **Observation and examination items**

Study period of patients

The study period for each patient is as follows;

| (1) Duration of study participation | Time from informed consent to the end of the observation period |
| --- | --- |
| (2) Observation period | Time from the date of registration to the last observation day (3 months after surgery) |

Observation/examination items and treatment information to be reported

The items to be investigated in this study are as follows:

At the time of registration

| Patient background | | |
| --- | --- | --- |
| Date of birth | (yyyy/mm/dd) | |
| Date of admission | (yyyy/mm/dd) | |
| Date of informed consent | (yyyy/mm/dd) | |
| Age at the time of consent |  | |
| Sex |  | |
| History of stroke | Subarachnoid hemorrhage, hemorrhagic stroke other than subarachnoid hemorrhage, ischemic stroke | |
| Comorbidity/lifestyle | Hypertension, diabetes mellitus, dyslipidemia, smoking habits | |
| Other concomitant medication | Antihypertensive, antidiabetic, dyslipidemic, anticoagulant (drug name) | |
| Pre-morbid mRS | 0, 1, 2, 3 | |
| Vital signs (on arrival at hospital) | | |
| Blood pressure | Systolic blood pressure, diastolic blood pressure (mmHg). | |
| Respiratory rate | (/min of times) | |
| Heart rate | (/min of times) | |
| Body temperature | (℃) | |
| Neurological findings on admission | | |
| Glasgow coma scale | E | 1, 2, 3, 4 |
|  | V | 1, 2, 3, 4, 5 |
|  | M | 1, 2, 3, 4, 5, 6 |
|  | Total score | 3, 4, 5, 6, 7, 8, 9, 10, 11, 12, 13, 14, 15 |
| Subarachnoid hemorrhage severity | | |
| WFNS class | I, II, III, IV, V | |
| Blood tests | | |
| Blood count | Hb, Plt | |
| Biochemical examination | Glu, LDL-chol, HbA1c, BNP, Alb | |
| Clotting marker | D-dimer | |
| Imaging studies | | |
| Modified Fisher grade | 0, 1, 2, 3, 4 | |
| Intraparenchymal brain hematoma | Yes (maximum diameter:), no | |

At the time of surgery

| Cerebral aneurysm information | | | | | |
| --- | --- | --- | --- | --- | --- |
| Prior treatment of the cerebral aneurysm | Yes (clipping, other), no | | | | |
| Site | ICA-paraclinoid, ICA-Pcom, ICA-ant.choroidal, ICA-bif, MCA M1, MCA bif, A1, Acom, distal ACA, VA, VA-PICA, BA trunk, BA-SCA, BA bif, PCA, others (ant. circulation), others (post. circulation) | | | | |
| Cerebral aneurysm geometry (angiography) | Maximal diameter (mm) ∗ does not include small bleb | | | | |
|  | Dome diameter (mm) | | | | |
|  | Neck diameter (mm) | | | | |
|  | Neck proximal mother vessel diameter (mm). | | | | |
|  | Incorporated branch (present, absent) | | | | |
| Characteristics of cerebral aneurysms | Thrombosed (present, absent), bleb (present, absent), others (). | | | | |
| Surgical procedures before cerebral aneurysm embolization | None, ventricular drainage, spinal drainage, decompressive craniectomy, hematoma evacuation, or other | | | | |
| Cerebral aneurysm embolization information | | | | | |
| Day of surgery | Sheath insertion time | | | (yyyy/mm/dd/HH/mm) | |
|  | Final intracranial angiographic time | | | (yyyy/mm/dd/HH/mm) | |
| Surgeon information | JSNET specialist information | Non-specialists, specialists, teaching physicians | | | |
|  | Number of surgical experiences for cerebral aneurysm | 0-9, 10-19, 20-39, 40-59, 60-99, 100  More than | | | |
| Surgical information | Approach site | | Inguinal, brachial, radial, carotid | | |
|  | Intraoperative heparin dose | | Total unit | | |
|  | ACT | | Control | | (sec) |
|  |  | | Maximum value | | (sec) |
|  | Use of a balloon-guiding catheter | | Yes, no | | |
|  | Use of balloon catheters other than neck modeling | | Yes (dilated and absent in blood vessels), no | | |
|  | Assistive techniques | | Use of DAC | | Yes, no |
|  |  |  | BAT | | Yes, no |
|  |  |  | SAT | | Yes, no |
|  |  |  | DCT | | Yes, no |
|  | Raymond Roy class | | I, II, IIIa, IIIb | | |
|  | Immediate postoperative embolization | | CO, NR, BF | | |
|  | Coil protrusion grade | | Yes (I, II, III), no | | |
|  | Intraoperative rupture | | Yes, no  (detailed description) | | |
|  | Intraoperative thrombus | | Yes, no  (detailed description)  Occluded vessels in the final angiography  Yes, no  Thrombus in final angiography  Yes, no | | |
|  | Other intraoperative complications | | Yes, no  (detailed description) | | |

Observation period (MRI: 6-48 h after surgery; CT, 48 h after surgery)

| MRI examination (6-48 h after surgery). | | | | | | | |
| --- | --- | --- | --- | --- | --- | --- | --- |
| Date and time of survey | | | (yyyy/mm/dd/HH/mm) | | | | |
| DWI | Presence of ischemic lesions | | | | | Yes, no | |
|  | Presence of symptomatic ischemic lesions | | | | | Yes (site, size), no | |
|  | Number of ischemic lesions in the area of the parent artery in the aneurysm | | | | | 0 – 4 mm |  |
|  |  | | | | | 5 – 9 mm |  |
|  |  | | | | | 10 – 14 mm |  |
|  |  | | | | | 15 mm or higher |  |
|  | Number of ischemic lesions other than the area of the aneurysm's parent artery | | | | | 0 – 4 mm |  |
|  |  | | | | | 5 – 9 mm |  |
|  |  | | | | | 10 – 14 mm |  |
|  |  | | | | | 15 mm or higher |  |
| MRA | Degree of aneurysm embolization | | | | | CO, NR, BF | |
|  | Newly occluded vessels | | | | | Yes, no | |
| CT examination (within 48 h after surgery) | | | | | | | |
| Date and time of survey | | | (yyyy/mm/dd/HH/mm) | | | | |
| Increased subarachnoid hemorrhage | | Yes, no | | | | | |
|  | | Modified Fisher grade | | | 0, 1, 2, 3, 4 | | |
| Increase in cerebral parenchymal blood species | | Yes, no | | | | | |
|  | | Size (maximum diameter) | | | | | |
| New parenchymal brain hematoma | | Yes (maximum diameter), no | | | | | |
| Increased hydrocephalus | | Yes, no | | | | | |
| Appearance of cerebral hemorrhage along tube tract  (only for cases with ventricular drainage) | | | | Yes (maximum diameter), no | | | |
| Surgical procedure after cerebral aneurysm treatment (during period after surgery to MRI scan), (multiple responses possible) | | | | | | | |
| Yes (ventricular drainage, spinal drainage, decompressive craniectomy, hematoma evacuation, cerebral aneurysm neck clipping, other), no | | | | | | | |
| Date of enforcement | | | | (yyyy/mm/dd) | | | |
| Antithrombotic therapy (during the period after surgery to MRI scan) | | | | | | | |
| Yes (detail), no | | | | | | | |
| Worsening of neurologic manifestations (during the period after surgery to MRI scan) | | | | | | | |
| Yes (disturbed consciousness, paralysis, sensory disturbance, aphasia, hemispatial neglect, others [details]), no | | | | | | | |

Observation period (14 days after surgery), acceptable range: ±2 days

| Day of investigation |
| --- |

| Day of investigation | (yyyy/mm/dd) |
| --- | --- |

| Subject information |
| --- |

| Continuous intravenous Nicardipine Hydrochloride therapy | Yes, no |
| --- | --- |
| IV Fasudil Hydrochloride Hydrate therapy | Yes, no |
| IV Ozagrel Sodium therapy | Yes, no |
| New cerebral infarction due to cerebral vasospasm | Yes, no |
| Endovascular treatment for symptomatic cerebral vasospasm. | Yes, no |
|  | Procedural details (), date, and time (). |

| Other antithrombotic therapies present |
| --- |
| Yes (detail), no |
| Safety information |

| Serious adverse event | Yes (if "yes", report details of "event/adverse event/occurrence of re-treatment"), no |
| --- | --- |
| Retreatment | Yes (if "yes", report details of "event/adverse event/occurrence of re-treatment"), no |

| CT |
| --- |

| Date and time of survey | (yyyy/mm/dd) |
| --- | --- |

| Increased subarachnoid hemorrhage | Yes, no | | |
| --- | --- | --- | --- |
|  | Modified Fisher grade | | 0, 1, 2, 3, 4 |
| Increase in cerebral parenchymal blood species | Yes (maximum diameter), no | | |
| New parenchymal brain hematoma | Yes (maximum diameter), no | | |
| Increased hydrocephalus | Yes, no | | |
| Appearance of cerebral hemorrhage along tube tract (only for cases with ventricular drainage) | | Yes (maximum diameter), no | |
| Surgical procedures after cerebral aneurysm treatment (multiple responses possible) | | | |
| Yes (ventricular drainage, spinal drainage, decompressive craniectomy, hematoma evacuation, cerebral aneurysm neck clipping, other), no | | | |

| Date of enforcement | (yyyy/mm/dd) |
| --- | --- |

| Worsening neurologic findings |
| --- |

| Yes (disturbed consciousness, paralysis, sensory disturbance, aphasia, hemispatial neglect, others (details)), no |
| --- |

Observation period (3 months after surgery) Acceptable range: ±14 days

| Day of investigation | |
| --- | --- |
| Day of investigation | (yyyy/mm/dd) |
| Subject information | |
| mRS | 0, 1, 2, 3, 4, 5, 6 |
| Shunt surgery for secondary hydrocephalus | Yes, no |
|  | Date (yyyy/mm/dd) |
|  | Ventriculoperitoneal shunt; lumbar peritoneal shunt; other |
| Safety information | |
| Serious adverse event | Yes (if "yes", report details of "event/adverse event/occurrence of re-treatment"), no |
| Retreatment | Yes (if "yes", report details of "event/adverse event/occurrence of re-treatment"), no |

Events, adverse events, and re-treatment occurrences (multiple responses possible)

| Event, adverse event, or retreatment | | |
| --- | --- | --- |
| Date of incident | (yyyy/mm/dd) | |
| Stroke events | | |
| Ruptured cerebral artery aneurysm | Treated aneurysm, otherwise (detail) | |
|  | WFNS Grade (I, II, III, IV, V) | |
|  | Retreatment: yes or no | |
|  | Date of retreatment | (yyyy/mm/dd) |
|  | Retreatment method | Craniotomy, endovascular treatment |
|  | Treatment outcome | CO, NR, BF |
| Other hemorrhagic stroke | Yes, no | |
|  | Details () | |
| Ischemic stroke | Yes, no | |
|  | Details () | |
| Other bleeding events | | |
| Significant hemorrhage by ISTH criteria  (Intracranial hemorrhage is described in the stroke event.) | Yes, no  Details () | |
| Other adverse events | Yes; details (), no | |

| Observation items    Time of implementation | Registration | Observation period | | | |  |
| --- | --- | --- | --- | --- | --- | --- |
|  | Preoperative | Intraoperative | Within 2 days after surgery | 14 days (±2 days) after surgery | 3 months (±14 days) after surgery | Time of event/event |
| Obtaining informed consent, registering  Allocation | ○ | − | − | − | − | − |
| Basic Information | ○ |  |  |  |  |  |
| Vital signs | ○ |  |  |  | − | − |
| Blood test | ○ |  | ○ | ○ |  |  |
| General blood chemistry | ○ |  | ○ | ○ |  |  |
| Clotting marker | ○ |  |  |  |  |  |
| Neurologic findings | ○ |  | ○ (during the period after surgery to MRI) | ○ | ○ | ○ |
| Daily life independence | ○ | − | − | − | ○ | ○ |
| Information on surgical technique | − | ○ | − | − | − | − |
| Images (MRI) | − | − | ○ (within 6-48 h after surgery) | − | − | − |
| Images (CT) | ○ | − | ○ (within 48 h after surgery) | ○ | − | − |
| Event | From time to time | | | | | − |
| Serious adverse event |  |  |  |  |  | − |
| Retreatment |  |  |  |  |  | − |

Event definitions, etc.

Antiplatelet agents administered before surgery that meet the exclusion criteria

　Ticlopidine, clopidogrel, prasugrel, cilostazol, aspirin

・Subarachnoid hemorrhage

　It is diagnosed by recognizing the hemorrhage in the subarachnoid space by head CT and MRI or by recognizing bloody cerebrospinal fluid by lumbar puncture.

Subarachnoid hemorrhage severity grading (WFNS grading)

　WFNS subarachnoid hemorrhage severity grading (1983) (see table below). The degree of subarachnoid hemorrhage on imaging is determined using a Modified Fisher grading system.

WFNS grade

| Severity | GCS scoring | Major focal neurologic symptoms (aphasia or hemiplegia) |
| --- | --- | --- |
| Grade I | 15 | Absent |
| Grade II | 14-13 | Absent |
| Grade III | 14-13 | Present |
| Grade IV | 12-7 | Present or absent |
| Grade V | 6-3 | Present or absent |

Glasgow coma scale (GCS, 1974)

| Large classification | Small classification | Score |
| --- | --- | --- |
| A. Eyes open | Spontaneously  To speech  To pain  No response | E4  E3  E2  E1 |
| B. Verbal responses | Oriented to time, person, and place  Confused  Inappropriate words  Incomprehensible sounds  No respnse | V5  V4  V3  V2  V1 |
| C. Best response to exercise | Obeys command  Moves to localized pain  Flex to withdraw from pain  Abnormal flexion  Abnormal extension  No response | M6  M5  M4  M3  M2  M1 |

Modified Fisher grading system

| 0 | No SAH or intraventricular hemorrhage |
| --- | --- |
| 1 | Focal or diffuse thin SAH without intraventricular hemorrhage |
| 2 | Focal or diffuse thin SAH with intraventricular hemorrhage |
| 3 | Focal or diffuse thick SAH without intraventricular hemorrhage |
| 4 | Focal or diffuse thick SAH with intraventricular hemorrhage |

・Measurement of cerebral aneurysm

1. Maximal diameter: Maximal diameter of cerebral aneurysm (excluding small projections and bleb)
2. Dome diameter: Cerebral aneurysm diameter parallel to the neck measured by the working angle (excluding small processes and blebs).
3. Neck diameter: Neck diameter measured at working angle

・Determination of the degree of embolization of cerebral aneurysms

　Based on the findings of the immediate postoperative angiography, the following classification is used to determine the results.

| Classification | Definitions |
| --- | --- |
| CO (complete) | Contrast does not flow into the aneurysm at all |
| NR (residual neck) | Contrast medium flow only near the neck |
| BF (partial obstruction) | Flow of contrast medium into the dome |

Raymond-Roy (2015)

| Classification | Definitions |
| --- | --- |
| Class I | Complete obliteration |
| Class II | Residual neck |
| Class IIIa | Residual aneurysm with contrast within coil interstices |
| Class IIIb | Residual aneurysm with contrast along aneurysm wall |

According to the findings of the immediate postoperative angiography, the degree of deviation of the coil is judged by the following classification.

Coil protrusion grade (2011)

| Classification | Definitions |
| --- | --- |
| Grade I | Loop or coil protrudes into the main lumen less than half of parent artery diameter. |
| Grade II | Coil protrudes into the main lumen exceeding more than half of parent artery diameter. |
| Grade III | Loop protrudes into the main lumen exceeding more than half of parent artery diameter. |

・Daily life independence

Modified Rankin Scale (mRS)

| Grade | Description |
| --- | --- |
| 0 | No symptoms at all |
| 1 | No significant disability despite symptoms; able to carry out all usual duties and activities |
| 2 | Slight disability; unable to carry out all previous activities, but able to look after own affairs without assistance |
| 3 | Moderate disability; requiring some help, but able to walk without assistance |
| 4 | Moderately severe disability; unable to walk without assistance and unable to attend to own bodily needs without assistance |
| 5 | Severe disability; bedridden, incontinent and requiring constant nursing care and attention |
| 6 | Dead |

Medical history and lifestyle habits.

|  | Definitions |
| --- | --- |
| Subarachnoid hemorrhage | Subarachnoid hemorrhage due to ruptured cerebral aneurysm not targeted in this study |
| Hemorrhagic stroke | Focal neurologic symptoms and confirmed intracranial hemorrhage by head CT or MRI |
| Ischemic stroke | Focal neurologic symptoms and confirmed cerebral infarction by head CT or MRI |
| High blood pressure | Any of the following criteria met within the three months prior to treatment: systolic blood pressure of 140 mmHg or higher, diastolic blood pressure of 90 mmHg or higher, or the use of antihypertensive medication to lower blood pressure. |
| Diabetes mellitus | Any of the following criteria met within the three months before treatment: fasting blood glucose of 126 mg/dL or higher, random blood glucose of 200 mg/dL or higher, HbA1c of 6.5% or higher, use of hypoglycemic agents, or a previous diagnosis of diabetes by a medical institution. |
| Dyslipidemia | Any of the following criteria met: LDL cholesterol of 140 mg/dL or higher, HDL cholesterol less than 40 mg/dL, triglycerides of 150 mg/dL or higher, or the use of lipid-lowering agents. |
| Smoking habit | Those who have a smoking habit of one or more cigarettes per day on average within 1 year before treatment. |

・Stroke events

　The following events occurring between surgery and 14 ± 2 days after surgery are defined as stroke events in this study:

|  | Definitions |
| --- | --- |
| Postoperative rupture of cerebral aneurysm | Symptomatic subarachnoid or intracerebral hemorrhage due to rupture　cerebral aneurysm after surgery diagnosed by CT or MRI. When asymptomatic or minimal headache occurs, the decision should be made with caution. WFNS Grade, presence or absence of retreatment, and treatment modalities should also be recorded. |
| Transient ischemic attack | Transient episodes of neurological dysfunction resulting from focal cerebral or retinal ischemia without evidence of acute infarction. Episodes of neurologic dysfunction should resolve within at least 24 h. |
| Ischemic stroke | Those with focal neurological symptoms lasting more than 24 h and confirmed ischemic by head CT or MRI. |
| Hemorrhagic stroke | Symptomatic subarachnoid and intracerebral hemorrhage without rupture of target cerebral aneurysms after surgery diagnosed by CT or MRI. This is not the case if the symptom is only a headache and not accompanied by other objective neurological symptoms. |

The above transient ischemic attack and ischemic stroke are combined to define a global ischemic event.

・Bleeding events

The following events that fall under "major bleeding" according to ISTH criteria are included in the "bleeding events" of this study.

1. Fatal bleeding

2. Symptomatic hemorrhage (intracranial, intrathecal, intraocular, retroperitoneal, intraarticular or intrapericardial, or intramuscular hemorrhage with muscular compartment syndrome) at critical sites or organs.

3. Bleeding resulting in a decrease in Hb level of 20 g/L or higher, bleeding leading to whole blood transfusion, or transfusion of two or more units of red blood cells.

・Cause of death

| Cerebral aneurysm death | Death from rupture of a cerebral aneurysm or from events directly related to a cerebral aneurysm |
| --- | --- |
| Death from stroke | Death from ischemic stroke and hemorrhagic stroke due to causes other than cerebral aneurysm |
| Other deaths | Death from causes other than cerebral aneurysm and stroke |
| Unknown | Death from unknown causes |

# **9.** **Discontinuation criteria**

- When it is judged impossible to continue the study for any reason, the physician-in-charge should discontinue the administration of the study drug and specify the date and timing of discontinuation or dropout, the reason for discontinuation or dropout, and course in the medical records and eCRF, as well as conduct necessary examinations at the time of discontinuation or dropout to evaluate efficacy and safety.

## 9-1. Discontinuation within the time from post-registration allocation to embolization

Discontinuation criteria

　　 1) When the research subject was asked to decline participation or withdraw consent

　　　2) When it is found that the eligibility criteria are not met after registration

　　 3) When the general condition deteriorates due to the re-rupture of the cerebral aneurysm, the surgery is discontinued.

4) When the entire study is discontinued

5) When the physician considers it appropriate to discontinue the study for other reasons

## 9-2. Discontinuation during the period from embolization to 3 months after surgery

Discontinuation criteria

1) When a participant has offered to decline research participation or withdrawn consent

2) When it is found that the eligibility is not satisfied after registration

3) When the entire study is discontinued

4) When the physician considers it appropriate to discontinue the study for other reasons

# **10. Handling of adverse events**

## **10-1. Response to patients when adverse events occur**

When adverse events are observed, the principal investigator or sub-investigator should promptly take appropriate measures and describe them in the medical records and eCRF without any discrepancy.

## **10-2. Reports on outbreaks of diseases**

When the following diseases (adverse events for which a causal relationship to the test drug or test procedure cannot be denied) occur and the principal investigator recognizes them, the principal investigator reports the fact to the manager of the participating medical organization and the accredited Clinical Research and Review Board and the Ministry of Health, Labour, and Welfare/PMDA within each period, as well as to the Clinical Research and Monitoring Committee of Tokyo Medical and Dental University:

| Unknown and known | Symptoms and conditions | Deadline for reporting |
| --- | --- | --- |
| Unknown | ・Death | 7 days |
|  | ・Diseases that may lead to death, etc. |  |
| Known | ・Death | 15 days |
|  | ・Diseases that may lead to death, etc. |  |
| Unknown | ① Inpatient hospitalization or prolongation of existing hospitalization  ② Disability  ③ Diseases that may lead to disability, etc.  ④ Diseases, etc. that are serious following the above and diseases that may lead to death or death  ⑤ Any congenital disease or anomaly in the offspring of a treated patient. |  |
| Other than the above (e.g., abnormal values, mild symptoms of disease, etc., mild but frequent, including bias in occurrence due to facilities) | | Periodic Report |

- The occurrence of diseases and their progress thereafter should be reported during periodic reporting.

# **11. Handling of the occurrence of non-compliance with the research protocol**

　The investigator shall promptly report to the manager of the medical institution when the clinical research is not in compliance with the Ministerial Authority or research protocol. (Report to the principal investigator, if known by the research sub-investigator) In the event of significant noncompliance, immediately hear the opinion of the accreditation committee. Nonconformance (Unified Form 7) will be reported periodically (Unified Form 5). In addition, the principal investigator will report to the manager of the participating medical organization and inform the research-representative physician. In case of significant noncompliance, the research representative physician should promptly seek the opinion of the accreditation committee. (Unified Form 7) Provide information to other investigators as soon as they know that the multicenter study is incompatible. Noncompliance should be reported in periodic reports (Unified Form 5).

# **12. Reporting and methods to administrators of medical institutions**

　The principal investigator will report the following items in writing to the manager of the medical institution (hospital director).

- The investigator shall promptly report to the manager of the participating medical organization if he/she knows that the clinical research is not in compliance with this Ministerial Authority or the research protocol (hereafter referred to as "incompatibility") (uniform forms 5 and 7).
- Principal investigator, research sub-investigator, and statistician.
- Persons who are listed in the study protocol and report to the manager about donations, manuscript writing, lectures, and other involvement in the work of the drug, marketed by the marketing author, used in the clinical study (Forms A-E).
- Promptly report to the manager if there is an opinion from the certified review board (uniform forms 4 and 13)
- Information provided to the principal investigator by the representative investigator in the multicenter study was promptly reported to managers.
- The primary endpoint report or clinical study report and summary will be submitted to the manager of the participating medical organization without delay when prepared or published (such a report).
- When the principal investigator (representative physician) submits the plan, the relevant information is reported to the manager for approval. (Initial application)
- Reports in case of illness within the prescribed period (Forms 8, 9, and 10; Form 2-1, 2-2; Form 6 for periodic illness reports; Form 3 for regular illness reports)
- A report to the manager within 30 days when a failure may occur and that disease, etc. (similar to serious adverse events) may occur (uniform forms 5 and 7).

# **13. Study completion, discontinuation, or interruption**

## **13-1. Completion of the study**

Upon completion, discontinuation, or interruption of the study at each institution, the principal investigator will submit the study completion, discontinuation, or interruption report to the head of the research institution ( head of the hospital) through the Clinical Trials Management Center. When the study is conducted in a multicenter setting, completion, discontinuation, or interruption reports will also be submitted to the representative study physician. In hospitals, the report shall be completed, discontinued, or interrupted, based on the report at the time of the implementation status survey at the end of each fiscal year.

After completion, discontinuation, or interruption is reported, the status is registered with the jRCT registry.

## **13-2. Discontinuation or interruption of research**

・ The principal investigator will consider whether or not to continue the study if any of the following is applicable.

1) When significant information on the quality, safety, and efficacy of the test drug is obtained.

2) When it is judged that it is difficult to recruit the study participants and to achieve the planned cases.

3) When the Certified Review Board determines that it is difficult to accept an order for a change in the implementation plan, etc.

If the Certified Review Board recommends or directs discontinuation, the study should be discontinued.

When the study is conducted at multiple institutions, the above items should be reviewed by the research representative physician or committee specified in the research protocol, and whether to continue the study should be considered.

・ If a decision is made to terminate or suspend a study, it should be promptly reported in writing to the head of the research institution (the head of the hospital) together with the clinical research review board. The head of the research institution (the head of the hospital) shall report to the Ministry of Health, Labour, and Welfare if the degree of non-compliance with the Medical Research Guidelines is discontinued due to its severity.

# **14. Statistical matter**

Details of statistical analyses will be specified in a separate statistical analysis plan.

## **14-1. Full analysis set**

　　The definitions of the analyzed population are as follows:

　Intention-to-treat (ITT) Group

All randomized populations will be included.

・Modified intention-to-treat (mITT) group

Patients in the ITT population excluded: ① patients who had never received a treatment either aspirin or placebo; ② patients who did not have any post-randomization data; and ③ patients who met the well-defined and objectively determinable criteria for selection and exclusion.

・Safety analysis set (Safety Analysis Set; SAS)

The study population will be randomized and will receive the treatment (aspirin or placebo).

## **14-2. Number of patients planned and rationale**

Target sample size 484

The rationale for setting the target number of patients :

The primary endpoints of this study were the rates of intraoperative thrombotic morbidity and symptomatic ischemic pathology on MRI-DWI (IRC). Rie et al reported^12^ a total of 8.8% incidence of thromboembolism in 159 patients with cerebral aneurysms who received intraoperative antiplatelet therapy and 17.6% in 102 patients who did not. In addition, at our institution and relevant institutions, based on the imaging findings obtained on MRI DWI on the day after the operation, in 27 patients who underwent endovascular aneurysm embolization for cerebral aneurysms, 14.8% (4 of 27 patients) showed ischemic lesion 15 mm or higher.

Therefore, the incidence of intraoperative thrombotic complications in the placebo-treated group and the incidence of symptomatic ischemic disease on MRI-DWI were assumed to be 15%. Assuming that aspirin treatment improves both by 10%, we assumed that the incidence of intraoperative thrombotic morbidity in the aspirin group and the incidence of symptomatic ischemic pathology on MRI-DWI would be 5% each. Under these assumptions, a two-sided significance level of 2.5% would result in a sample size of 460 patients (230 patients in each group), requiring 90% power for each intergroup comparison of the endpoints. The target sample size was 484 patients (242 in each group) with a dropout rate of approximately 5%.

## **14-3. Statistical analysis**

**Analysis of the primary endpoint**

1) Incidence of intraoperative thrombotic complications

2) Incidence of symptomatic ischemic involvement in MRI-DWI (IRC).

The superiority of the aspirin-treated group to the placebo-treated group will be tested in mITT using Cochran-Mantel-Haenszel (CMH test stratified by age (≧65 years, <65 years), sex (men, women), WFNS grade (I-III, IV-V), and modified Fisher grade (0-2, 3-4. Considering the multiplicity of tests, the two-sided significance level in the comparison between the groups for each endpoint was 2.5 %. The number and percentage of patients with events were calculated for each group, and the difference in the percentages between the two groups (aspirin group vs. placebo group) was calculated with a 97.5% confidence interval.

**Analysis of the key secondary endpoints**

1) Incidence of all bleeding events within 14 days of onset

2) Incidence of global cerebral ischemic events in the first 14 days after MRI radiography after surgery

For mITT, the number and percentage of patients with events will be calculated in each group, and the difference in the percentage between the two groups (aspirin-placebo group) and the 95% confidence interval will be calculated. If the upper limit of the confidence interval was less than 10%, non-inferiority was demonstrated. Comparisons between the groups for each endpoint will be based on the closed test procedure described above.

**Analysis of secondary endpoints**

1) Incidence of symptomatic ischemic involvement in MRI-DWI (INV).

2) mRS at 3 months

Group comparisons will be performed in the mITT using CMH tests stratified by age (≧65 years, <65 years), sex (men, women), WFNS grade (I-III, IV-V), and modified Fisher grade (0-2, 3-4. The two-sided significance level was set at 5 %. In comparing the groups, mRS is defined as the percentage of patients scoring 2 or less at 3 months. In addition, the number and percentage of patients with events will be calculated for each group, and the difference in the percentage between the two groups (aspirin and placebo groups) and the 95% confidence interval will be calculated. In addition, for the mRS, the number and percentage of patients with an mRS score ranging from 0 to 6 points in the groups were obtained, and the proportional odds model was used to obtain the odds ratio and 95% confidence interval.

**Analysis of exploratory endpoints**

1) Ischemic involvement (number and size) in MRI-DWI

Summary numbers and sizes of individual groups will be calculated for the mITT and compared between groups.

2) Incidence of cerebral hemorrhage along tube tract of ventricular drainage in CT in the first 14 days after surgery.

　　　For mITT, the number and percentage of patients with events will be calculated in each group, and the difference in the percentage between the two groups (aspirin-placebo group) and the 95% confidence interval will be calculated.

**Analysis of safety endpoints**

1) Adverse event rates

2) Incidence of adverse reactions

The numbers and percentages of patients in each group are calculated for SAS.

**Interim analysis**

No interim analysis will be performed in this study.

# **15. Monitoring**

Regular monitoring will be conducted twice a year to ensure that the study is conducted safely and in accordance with the protocol and that the data are accurately collected. Monitoring was based on eCRF-completed data collected in the data center and centrally monitored by the data center, research secretariat, and research representative physicians. Monitoring was not performed during site visits, and reconciliation was conducted with source documents. The data center submits the prepared periodic monitoring report to the research office and the research representative physician. The purpose of periodic monitoring is to increase the scientific ethics of trials by feeding back on problems and is not intended to identify problems in research or institutions. Therefore, research representative physicians review periodic monitoring reports, share problems with researchers at participating institutions, and strive to improve them.

# **16 Ethics**

## **16-1. Declaration of Helsinki and the Clinical Research Act.**

The study will be conducted in compliance with the Declaration of Helsinki (October 2013) and the Clinical Research Act (April 14, 2017).

## **16-2. Consideration for human rights (protection of privacy)**

　　To protect the privacy of research participants, identification codes that do not include elements of personal information are used to identify individual research participants, and even in the publication of research results, information that can identify the identity is protected without publication.

## **16-3. Consideration for safety and disadvantage**

　The aspirin used in this study is off-label for the study population, but it does not suffer any apparent disadvantages compared with daily insurance practice. Although the risk of adverse events arises with treatment, participation in this study does not increase these risks relative to routine practice.

There was no apparent disadvantage to the study participants assigned to the placebo group because they will be treated similarly to the existing treatment. If necessary, the emergency key is opened to confirm the treatment assignment.

Questions related to the study can be consulted at any time, and discontinuation can be offered at any time if the patient does not want to continue the study. In addition, appropriate measures should be taken when adverse reactions that may be related to this study occur, even after the discontinuation or completion of the study.

# **17.** **Cost burden for the study participants**

Because medical care for the research participant under study is within the scope of usual medical care, the medical cost burden of the insurance medical treatment division arises for the research participant. Additionally, the Research Secretariat incurred the cost of the study treatment (aspirin or placebo).

# **18. Health Damage Compensation and Insurance Subscription**

We have clinical research insurance covering this study. For the aspirin used in this study, compensation was provided in the event of an unknown adverse reaction, excluding known adverse reactions specified in the instructions distributed to the research participants.

## **18-1. Compensation for health damage**

◆ The costs for treatment of health hazards at Tokyo Medical and Dental University Hospital and related facilities are to be addressed in accordance with the Principles of Cost Burden in Applications for Voluntary Clinical Research, Unapproved Drugs, which are listed on the Website of the Center for Clinical Trial Management, Tokyo Medical and Dental University Hospital.

## **18-2. Purchase of compensation insurance and liability insurance**

In preparation for compensation and liability, the research investigator and sub-investigator will join the compensation and liability insurance.

# **19. Handling of Personal Information**

To protect the privacy of the research participants, the research participants’ identification codes is used to identify the individual research participants, and their personal information was protected. Even when research results are published, information that can identify the person under study is protected.

Furthermore, the samples and information obtained from this study may be provided to other research institutions after obtaining approval from the Certified Review Board of Tokyo Medical and Dental University, with the protection of the participant’s personal information.

# **20. Storage of records**

　　Person in charge of recordkeeping: Kazutaka Sumita

Department of Endovascular Surgery, Tokyo Medical and Dental University Hospital

Location: Yushima 1-5-45, Bunkyo District, Tokyo

Telephone number (direct): 03-3813-4088

The person responsible for the preservation of records shall retain copies of essential documents and application documents related to the conduct of research, notifications from the director of the hospital, copies of various application forms and reports, copies of research object identification code lists, consent forms, eCRF, and other documents or records required to ensure data reliability. Such documents and records shall be disposed of 10 years after publication of the research.

# **21. Enrollment of study plans and publication of study results**

The outline of the research (name, objective, method, implementation system, selection policy of the participant, etc.) shall be registered in the Clinical Research Protocol and Publication System of Research Outline (jRCT) https://jrct.niph.go.jp prior to its implementation, updated as appropriate according to the changes in the research protocol and the progress of the research, and if the research is completed, the results of the research shall be registered without delay.

The primary results will be presented at international conferences or in English.

# **22. System for conducting research**

| Role | Name | Title | Medical institutions | Name of the department | Address | Contact |
| --- | --- | --- | --- | --- | --- | --- |
| Principle Investigator | Kazutaka Sumita | Professor | Tokyo Medical and Dental University | Endovascular surgery | 1-5-45, Yushima, Bunkyo-ku, Tokyo | 03-3813-6111 |
| Physicians in charge of the research | Hirai Sakyo | Junior associate professor | Tokyo Medical and Dental University | Endovascular surgery | 1-5-45, Yushima, Bunkyo-ku, Tokyo | 03-3813-6111 |
| Investigator | Keigo shigeta | Chief physician | National Hospital Organization Disaster Medical Center | Neurosurgery | 3256, Midori-cho, Tachikawa-shi, Tokyo | 042-526-5511 |
| Investigator | Sato Yohei | Director | Japanese Red Cross Musashino Hospital | Neurosurgery | 1-26-1, sakaiminami-cho, Musashino-shi, Tokyo | 0422-32-3111 |
| Investigator | Shin Hirota | Director | Tsuchiura Kyodo General Hospital | Neurosurgery | 4-1-1, Ootsuno, Tsuchiura-shi, Ibaraki | 029-830-3711 |
| Investigator | YoshikazuYoshino | Professor | Jichi Medical University Saitama Medical Center | Neuroendovascular surgery | 1-847, Amanuma-cho, Omiya-ku, Saitama-shi, Saitama | 048-647-2111 |
| Investigator | Jun karakama | Director | Ome Medical Center | Neurosurgery | 4-16-5, Higashi-Ome, Ome-shi, Tokyo | 0428-22-3191 |
| Investigator | Ishii Yosuke | Director | Kanto Rosai Hospital | Neurosurgery | 1-1, Kizuki-Sumiyoshi-cho, Nakahara-ku, Kawasaki-shi, Kanagawa | 044-411-3131 |
| Investigator | Mutsuya Hara | Director | Tokyo Metropolitan Toshima Hospital | Neurosurgery | 33-1, Sakae-cho, Itabashi-ku, Tokyo | 03-5375-1234 |
| Investigator | Toshihiro Yamamura | Director | JA Toride Medical Center | Neurosurgery | 2-1-1, Hongo, Toride-shi, Ibaraki | 0297-74-5551 |
| Investigator | Motoshige Yamashina | Chief physician | Soka Municipal Hospital | Neurosurgery | 2-21-1, Soka, Soka-shi, Saitama | 048-946-2200 |
| Investigator | Shogo imae | Director | Fujiyoshida Municipal Hospital | Neurosurgery | 7-11-1, Kamiyoshidahigashi, Fujiyoshida-shi, Yamanashi | 0555-22-4111 |
| Investigator | Kana Sawada | Chief physician | Tokyo Bay and Urayasu Ichikawa Medical Center | Neurosurgery | 3-4-32, Todaijima, Urayasu-shi, Chiba | 047-351-3101 |
| Investigator | Yoshiki Obata | Director | Tokyo Kita Medical Center | Neurosurgery | 4-17-56, Akabanedai, Kita-ku, Tokyo | 03-5963-3311 |
| Investigator | Ishiwada Tadahiro | Chief physician | Shioda Memorial Hospital | Neurosurgery | 550-1, Koori, Nagara-machi, Chosei-gun, Chiba | 0475-35-0099 |
| Investigator | Naoki Taira | Director | Shuuwa General Hospital | Neurosurgery | 1200, Yaharashinden, Kasukabe-shi, Saitama | 048-737-2121 |
| Investigator | Mizoue Tatsuya | Director | Seisuikai Kajikawa Hospital | Neurosurgery | 1-1-23, Higashisendamachi, Naka-ku, Hiroshima-shi, Hiroshima | 082-249-6411 |
| Investigator | Masahiro Indo | Director | Higashiyamato Hospital | Neurosurgery | 1-13-12, Minamimachi, Higashiyamato-shi, Tokyo | 042-562-1411 |
| Investigator | Sato Hiroaki | Director | Tokyo Metropolitan Police Hospital | Neuroendovascular surgery | 4-22-1, Nakan, Nakano-ku, Tokyo | 03-5343-5611 |
| Investigator | Yukiko Enomoto | Associate professor | Gifu University Hospital | Neurosurgery | 1-1, Yanagito, Gifu-shi, Gifu | 058-230-6000 |
| Investigator | Masato Inoue | Director | Center Hospital of the National Center for Global Health and Medicine | Neurosurgery | 1-21-1, Toyama, Shinjuku-ku, Tokyo | 03-3202-7181 |
| Investigator | Mochida Hidetoshi | Director | Asahi General Hospital | Neurosurgery | I 1326, Asahi-shi, Chiba | 0479-63-8111 |
| Investigator | Yasushi Takagi | Professor | Tokushima University Hospital | Neurosurgery | 2-50-1, Kuramoto-cho, Tokushima-shi, Tokushima | 088-631-3111 |
| Investigator | Sakamoto Makoto | Associate professor | Tottori University Hospital | Neurosurgery | 36-1, Nishi-cho, Yonago-shi, Tottori | 0859-33-1111 |
| Investigator | Koichi Arimura | Junior associate professor | Kyushu University Hospital | Neurosurgery | 3-1-1, Maidashi, Higashi-ku, Fukuoka | 092-641-1151 |
| Investigator | Kouichi Misaki | Junior associate professor | Kanazawa University Hospital | Neurosurgery | 13-1, Takaramachi, Kanazawa-shi, Ishikawa | 076-265-2000 |
| Investigator | Takayuki Amano | Director | Nagoya Tokushukai General Hospital | Neurosurgery | 2-52, Kozoji-cho Kita, Kasugai-shi, Aichi | 0568-51-8711 |
| Investigator | Nanto Masataka | Junior associate professor | Kyoto Prefectural University of Medicine | Neurosurgery | 465, Kajii-cho, Hirokoji-agaru, Kawaramachidori, Kamigyo-ku, Kyoto-shi, Kyoto | 075-251-5111 |
| Investigator | Kenichi Matsumoto | Director | Saga Prefectural Hospital Koseikan | Neurosurgery | 400, Nakahara, Kase-cho, Saga-shi, Saga | 0952-24-2171 |
| Investigator | Nobukuni Murakami | Director | Japanese Red Cross Society Kyoto Daini Hospital | Neurosurgery | 355-5, Haruobi-cho, Marutamachi agaru, Kamanza-dori, Kamigyo-ku, Kyoto-shi | 075-231-5171 |
| Investigator | Masataka Takeuchi | Director | Seisho Hospital | Neurosurgery | 1-16-35, Oogimachi, Odahara-shi, Kanagawa | 0465-35-5773 |
| Investigator | Yoshiki Hanaoka | Associate professor | Shinshu University Hospital | Neurosurgery | 3-1-1 Asahi, Matsumoto-shi, Nagano | 0263-35-4600 |
| Investigator | Shinichi Yoshimura | Professor | Hyogo University Hospital | Neurosurgery | 1-1 Mukogawa-cho, Nishinomiya-shi, Hyogo | 0798-45-6111 |
| Investigator | Junichi Miyamoto | Director | Kyoto Saiseikai Hospital | Neurosurgery | 101, Shimouchida, Shimokaiinji, Nagaokakyo-shi, Kyoto | 075-955-0111 |
| Investigator | Kohyama Shinya | Professor | Saitama Medical University International Medical Center | Endovascular Neurosurgery | 1397-1, Yamane, Hidaka-shi, Saitama | 042-984-4111 |
| Investigator | So Tokunaga | Director | National Hospital Organization Kyushu Medical Center | Neuroendovascular surgery | 1-8-1, Jigyohama, Chuo-ku, Fukuoka | 092-852-0700 |
| Investigator | Toshihiro Yamauchi | Director | Chiba Emergency and Psychiatric Medical center | Neurosurgery | 3-32-1, Isobe, Mihama-ku, Chiba-shi, Chiba | 043-279-2211 |
| Investigator | Toshio Higashi | Professor | Fukuoka University Chikushi Hospital | Neurosurgery | 1-1-1, Zokumyoin, Chikushino-shi, Fukuoka | 092-921-1011 |
| Investigator | Masaru Hirohata | Professor | Kurume University Hospital | Neurosurgery | 67, Asahi-cho, Kurume-shi, Fukuoka | 0942-35-3311 |
| Investigator | Masafumi Morimoto | Director | Yokohama Shintoshi Neurosurgical Hospital | Neurosurgery | 433, Edacho, Aoba-ku, Yokohama-shi, Kanagawa | 045-911-2011 |
| Investigator | Michihiro Hayasama | Director | Kimitsu Chuo Hospital | Neurosurgery | 1010, Sakurai, Kisarazu-shi, Chiba | 0438-36-1071 |
| Investigator | Tomoji Takigawa | Associate professor | Dokkyo Medical University Saitama Medical Center | Neurosurgery | 2-1-50, Minamikoshigaya, Koshigaya-shi, Saitama | 048-965-1111 |
| Investigator | Kazuo Hanakawa | Director | Tokyo Metropolitan Bokutoh Hospital | Neurosurgery | 4-23-15, Kotobashi, Sumida-ku, Tokyo | 03-3633-6151 |
| Investigator | Yukihiro Hidaka | Director | Saitama Sekishinkai Hospital | Neuroendovascular surgery | 2-37-20, Irumagawa, Sayama-shi, Saitama | 04-2953-6611 |
| Investigator | Hirofumi Nakatomi | Professor | Kyorin University Hospital | Neurosurgery | 6-20-2, Shinkawa, Mitaka-shi, Tokyo | 0422-47-5511 |
| Pharmacist (preparation of test drug) | Kazuhiko Arakawa | Pharmacist | Tokyo Medical and Dental University Hospital | Hospital Pharmacy | 1-5-45, Yushima, Bunkyo-ku, Tokyo | 03-5803-5601 |
| Pharmacist (preparation of test drug) | Yu Takahashi | Pharmacist | Tokyo Medical and Dental University Hospital | Hospital Pharmacy | 1-5-45, Yushima, Bunkyo-ku, Tokyo | 03-5803-5601 |
| Study manager | Megumi Ishiguro | Associate professors | Tokyo Medical and Dental University | Health Sciences R&D Center | 1-5-45, Yushima, Bunkyo-ku, Tokyo | 03-3813-6111 |
| Data manager | Pariko Yorozu | URA | Tokyo Medical and Dental University | Health Sciences R&D Center | 1-5-45, Yushima, Bunkyo-ku, Tokyo | 03-3813-6111 |
| Monitoring manager | Makoto Ishii | URA | Tokyo Medical and Dental University | Health Sciences R&D Center | 1-5-45, Yushima, Bunkyo-ku, Tokyo | 03-3813-6111 |
| Coordination office | Emi yoshida | URA | Tokyo Medical and Dental University | Health Sciences R&D Center | 1-5-45, Yushima, Bunkyo-ku, Tokyo | 03-3813-6111 |
| Statistical analysis manager | Akihiro Hirakawa | Professor | Tokyo Medical and Dental University | Clinical Biostatistics | 1-5-45, Yushima, Bunkyo-ku, Tokyo | 03-3813-6111 |
| Statistical analyst | Hiroyuki Sato | Assistant professor | Tokyo Medical and Dental University | Clinical Biostatistics | 1-5-45, Yushima, Bunkyo-ku, Tokyo | 03-3813-6111 |
| Statistical analyst | Ryoichi hanazawa | Researcher | Tokyo Medical and Dental University | Clinical Biostatistics | 1-5-45, Yushima, Bunkyo-ku, Tokyo | 03-3813-6111 |
| Statistical analyst | Seiji Sasaki | Researcher | Tokyo Medical and Dental University | Clinical Biostatistics | 1-5-45, Yushima, Bunkyo-ku, Tokyo | 03-3813-6111 |
| Independent review committee members | Ooyama Jun | Junior associate professor | Tokyo Medical and Dental University Hospital | Radiology | 1-5-45, Yushima, Bunkyo-ku, Tokyo | 03-3813-6111 |
| Independent review committee members | Kota Yokoyama | Assistant professor | Tokyo Medical and Dental University Hospital | Radiology | 1-5-45, Yushima, Bunkyo-ku, Tokyo | 03-3813-6111 |
| Independent review committee members | Shoko Hara | Assistant professor | Tokyo Medical and Dental University Hospital | Neurosurgery | 1-5-45, Yushima, Bunkyo-ku, Tokyo | 03-3813-6111 |

Consultation service

From 10:00 to 16:00 on weekdays to Kazutaka Sumita, principal investigator, to Tokyo Medical and Dental University Hospital on Saturdays, Sundays, and Holidays.

# **23. Research funding and conflicts of interest**

　The Certified Review Board confirms that there is no "possible conflict of interest" in the planning, implementation, and reporting of this study that affects the interpretation of the results and results of the study and that the conduct of the study does not compromise the rights and interests of the research participants.

# **24. Changes in the research protocol**

　When necessary, the principal investigator will revise the protocol after obtaining approval from the research coordinating committee. Except for minor cases, in the case of revisions, the Certified Review Board shall undergo a repeat review of the contents of the revisions and the reasons for the revisions, and obtain approval.

# **25. Reference lists**

1. Molyneux A, Kerr R, Stratton I, et al. International Subarachnoid Aneurysm Trial (ISAT) of neurosurgical clipping versus endovascular coiling in 2143 patients with ruptured intracranial aneurysms: a randomised trial. Lancet 2002;360:1267-1274
2. Molyneux AJ, Kerr RS, Yu LM, et al. International subarachnoid aneurysm trial (ISAT) of neurosurgical clipping versus endovascular coiling in 2143 patients with ruptured intracranial aneurysms: a randomised comparison of effects on survival, dependency, seizures, rebleeding, subgroups, and aneurysm occlusion. Lancet 2005;366:809-817
3. Park HK, Horowitz M, Jungreis C, et al. Periprocedural morbidity and mortality associated with endovascular treatment of intracranial aneurysms. AJNR Am J Neuroradiol 2005;26:506-514
4. Zheng Y, Liu Y, Leng B, et al. Periprocedural complications associated with endovascular treatment of intracranial aneurysms in 1764 cases. J Neurointerv Surg 2016;8:152-157
5. Hwang G, Jung C, Park SQ, et al. Thromboembolic complications of elective coil embolization of unruptured aneurysms: the effect of oral antiplatelet preparation on periprocedural thromboembolic complication. Neurosurgery 2010;67:743-748; discussion 748
6. Edwards NJ, Jones WH, Sanzgiri A, et al. Antiplatelet therapy for the prevention of peri-coiling thromboembolism in high-risk patients with ruptured intracranial aneurysms. J Neurosurg 2017;127:1326-1332
7. Almekhlafi MA, Al Sultan AS, Kuczynski AM, et al. Antiplatelet therapy for prevention of thromboembolic complications in coiling-only procedures for unruptured brain aneurysms. J Neurointerv Surg 2020;12:298-302
8. Skukalek SL, Winkler AM, Kang J, et al. Effect of antiplatelet therapy and platelet function testing on hemorrhagic and thrombotic complications in patients with cerebral aneurysms treated with the pipeline embolization device: a review and meta-analysis. J Neurointerv Surg 2016;8:58-65
9. Fujii Y, Takeuchi S, Sasaki O, et al. Hemostasis in spontaneous subarachnoid hemorrhage. Neurosurgery 1995;37:226-234
10. Juvela S, Siironen J. D-dimer as an independent predictor for poor outcome after aneurysmal subarachnoid hemorrhage. Stroke 2006;37:1451-1456
11. Cognard C, Pierot L, Anxionnat R, et al. Results of embolization used as the first treatment choice in a consecutive nonselected population of ruptured aneurysms: clinical results of the Clarity GDC study. Neurosurgery 2011;69:837-841; discussion 842
12. Ries T, Buhk JH, Kucinski T, et al. Intravenous administration of acetylsalicylic acid during endovascular treatment of cerebral aneurysms reduces the rate of thromboembolic events. Stroke 2006;37:1816-1821
13. Van den Bergh WM, Kerr RS, Algra A, et al. Effect of antiplatelet therapy for endovascular coiling in aneurysmal subarachnoid hemorrhage. Stroke 2009;40:1969-1972
14. Shimamura N, Naraoka M, Matsuda N, et al. Use of Preprocedural, Multiple Antiplatelet Medications for Coil Embolization of Ruptured Cerebral Aneurysm in the Acute Stage Improved Clinical Outcome and Reduced Thromboembolic Complications without Hemorrhagic Complications. World Neurosurg 2020;133:e751-e756
15. Kung DK, Policeni BA, Capuano AW, et al. Risk of ventriculostomy-related hemorrhage in patients with acutely ruptured aneurysms treated using stent-assisted coiling. J Neurosurg 2011;114:1021-1027
16. Dorhout Mees SM, van den Bergh WM, Algra A, et al. Antiplatelet therapy for aneurysmal subarachnoid haemorrhage. Cochrane Database Syst Rev 2007:Cd006184
17. Bodily KD, Cloft HJ, Lanzino G, et al. Stent-assisted coiling in acutely ruptured intracranial aneurysms: a qualitative, systematic review of the literature. AJNR Am J Neuroradiol 2011;32:1232-1236
