# Supplementary material for: Study protocol of the ASTOP trial: A multicenter, randomized, double-blind, placebo-controlled trial of presurgical aspirin administration for the prevention of thromboembolic complications of coil embolization for ruptured aneurysms
Source: PLoS One. 2024 Sep 26;19(9):e0310906. doi: 10.1371/journal.pone.0310906 (PMC11426478; doi:10.1371/journal.pone.0310906)
Supplement: S3 File — (PDF) [file pone.0310906.s003.pdf]

Reference number	NR2021-006

Uniform format 4

09/17/2021

Notification of Examination Results

<u>Principal investigator</u>

Tokyo Medical and Dental University Hospital

Department of Endovascular surgery, Professor

Kazutaka Sumita

<u>Certified Clinical Research Review Board</u>

Chairman of the Clinical Research Review Committee, Tokyo Medical and DentalUniversity

Regional Health and Welfare Bureau: Kanto-Shinetsu Regional Bureau of Health and Welfare

We would like to notify you of the results of your review request as follows:

jRCT Number	jRCTs031210421
Research Name	A multicenter, randomized, double-blind, placebo-controlled trial of pre-surgical aspirin administration for the prevention of thromboembolic complications of coil embolization for ruptured aneurysms　(ASTOP study)
Examination items	The suitability of conducting clinical research<br>（New Review Request Form　Date: 08/20/2021）
Examination category	Committee Review（09/16/2021）
Examination results	approve

Approval Documents

Document Title	Creation date	Version
Implementation Plan (Ministerial Ordinance Form No. 1)	08/18/2021	1
Research plan	08/18/2021	1
Information document, consent form	08/18/2021	1
Documents outlining the pharmaceuticals, etc.	March, 2021	
Procedures for responding to outbreaks of illness, etc.	08/16/2021	
Monitoring Procedures	08/16/2021	
Conflict of Interest Management Plan (Form A)	08/12/2021	
Conflict of Interest Management Plan (Form E)	Number of institutions: 16	
List of research physicians	Number of institutions: 16	
