# Supplementary material for: Study protocol of the ASTOP trial: A multicenter, randomized, double-blind, placebo-controlled trial of presurgical aspirin administration for the prevention of thromboembolic complications of coil embolization for ruptured aneurysms
Source: PLoS One. 2024 Sep 26;19(9):e0310906. doi: 10.1371/journal.pone.0310906 (PMC11426478; doi:10.1371/journal.pone.0310906)
Supplement: S4 File — (PDF) [file pone.0310906.s004.pdf]

| Reference number | NR2021-006 |
|---|---|

Uniform format 4

03/22/2024

# Notification of Examination Results

Principal investigator
Tokyo Medical and Dental University Hospital
Department of Endovascular surgery, Professor
Kazutaka Sumita

Certified Clinical Research Review Board
Chairman of the Clinical Research Review Committee, Tokyo Medical and DentalUniversity
Regional Health and Welfare Bureau: Kanto-Shinetsu Regional Bureau of Health and Welfare

We would like to notify you of the results of your review request as follows:

| jRCT Number | jRCTs031210421 |
|---|---|
| Research Name | A multicenter, randomized, double-blind, placebo-controlled trial of pre-surgical aspirin administration for the prevention of thromboembolic complications of coil embolization for ruptured aneurysms (ASTOP study) |
| Examination items | Proper continuation of clinical research<br>Changes in Implementation Plan<br>（Change Review Request Form　Date: 02/26/2024） |
| Examination category | Committee Review（03/21/2024） |
| Examination results | approve |

Approval Documents

| Document Title | Creation date | Version |
|---|---|---|
| Implementation Plan (Ministerial Ordinance Form No. 1) | 02/26/2024 | |
| Research plan | 02/16/2024 | 20 |
| Information document, consent form | 02/16/2024 | 18 |
| Conflict of Interest Management Plan | 02/05/2024 | |
| List of research physicians | 02/05/2024 | |
| Others | | |
| Research plan_ Change comparison table | 02/16/2024 | |
| Informed consent form_ Change comparison table | 02/16/2024 | |
| Notification of changes to clinical research plan | 02/26/2024 | |
