# Supplementary material for: Study protocol of the ASTOP trial: A multicenter, randomized, double-blind, placebo-controlled trial of presurgical aspirin administration for the prevention of thromboembolic complications of coil embolization for ruptured aneurysms
Source: PLoS One. 2024 Sep 26;19(9):e0310906. doi: 10.1371/journal.pone.0310906 (PMC11426478; doi:10.1371/journal.pone.0310906)
Supplement: S5 File — (PDF) [file pone.0310906.s005.pdf]

List of research facilities

Department of Endovascular surgery, Tokyo Medical and Dental University, Tokyo, Japan; Department of Neurosurgery, National Hospital Organization Disaster Medical Center, Tokyo, Japan; Department of Neurosurgery, Japanese Red Cross Musashino Hospital, Tokyo, Japan; Department of Neurosurgery, Tsuchiura Kyodo General Hospital, Ibaraki, Japan; Department of Neurosurgery, Ome Medical Center, Tokyo, Japan; Department of Neurosurgery, Soka Municipal Hospital, Saitama, Japan; Department of Neurosurgery, Tokyo Metropolitan Toshima Hospital, Tokyo, Japan; Department of Neurosurgery, JA Toride Medical Center, Ibaraki, Japan; Department of Neurosurgery, Fujiyoshida Municipal Hospital, Yamanashi, Japan; Department of Neurosurgery, Shuuwa General Hospital, Saitama, Japan; Department of Neurosurgery, Tokyo Bay Urayau Ichikawa Medical Center, Chiba, Japan; Department of Neurosurgery, Shioda Memorial Hospital,Chiba,Japan; Department of Neurosurgery, Tokyo Kita Medical Center, Tokyo, Japan; Department of Neuroendovascular surgery, Jichi Medical University Saitama Medical Center, Saitama, Japan; Department of Neurosurgery, Kanto Rosai Hospital, Kanagawa, Japan; Department of Neurosurgery, Seisuikai Kajikawa Hospital, Hiroshima, Japan; Department of Neurosurgery, Higashiyamato Hospital, Tokyo, Japan; Department of Neuroendovascular surgery, Tokyo Metropolitan Police Hospital, Tokyo, Japan; Department of Neurosurgery, Gifu University Graduate School of Medicine, Gifu, Japan; Department of Neurosurgery, Center Hospital of the National Center for Global Health and Medicine, Tokyo, Japan; Department of Neurosurgery, Asahi General Hospital, Chiba, Japan; Division of Neurosurgery, Department of Brain and Neurosciences, Faculty of Medicine, Tottori University; Department of Neurosurgery, Tokushima University Hospital, Tokushima, Japan; Department of Neurosurgery, Graduate School of Medical Sciences, Kyushu University, Fukuoka, Japan; Department of Neurosurgery, Kanazawa University Hospital, Kanazawa, Japan; Department of Neurosurgery, Nagoya Tokushukai General Hospital, Aichi. Japan; Department of Neurosurgery, Graduate School of Medical Science, Kyoto Prefectural University of Medicine, Kyoto, Japan; Department of Neurosurgery, Saga Prefectural Hospital Koseikan, Saga, Japan; Department of Neurosurgery, Japanese Red Cross Society Kyoto Daini Hospital, Kyoto, Japan;

Department of Neurosurgical, Seisho Hospital, Kanagawa, Japan;
Department of Neurosurgery, Shinshu University Hospital, Nagano, Japan;
Department of Neurosurgery, Hyogo Medical University Hospital, Hyogo, Japan; Junichi Miyamoto, Department of Neurosurgery, Kyoto Saiseikai Hospital, Kyoto, Japan;
Department of Endovascular Neurosurgery, Saitama Medical University International Medical Center, Saitama, Japan;
Department of Neuroendovascular surgery, National Hospital Organization Kyushu Medical Center, Fukuoka, Japan;
Department of Neurosurgery, Chiba Emergency and Psychiatric Medical center, Chiba, Japan; Department of Neurosurgery, Fukuoka University Chikushi Hospital, Fukuoka, Japan;
Department of Neurosurgery, Kurume University Hospital, Fukuoka, Japan;
Department of Neurosurgery, Yokohama Shintoshi Neurosurgical Hospital, Kanagawa, Japan; Department of Neurosurgery, Kimitsu Chuo Hospital, Chiba, Japan;
Department of Neurosurgery, Dokkyo Medical University Saitama Medical Center, Saitama, Japan;
Department of Neurosurgery, Tokyo Metropolitan Bokutoh Hospital, Tokyo, Japan;
Department of Neurosurgery, Saitama Sekishinkai Hospital, Saitama, Japan;
Department of Neurosurgery, Kyorin University Hospital, Tokyo, Japan
